# Proteome changes in platelets activated by arachidonic acid, collagen, and thrombin

**DOI:** 10.1186/1477-5956-8-56

**Published:** 2010-11-12

**Authors:** Pavel Májek, Zuzana Reicheltová, Jana Štikarová, Jiří Suttnar, Alžběta Sobotková, Jan E Dyr

**Affiliations:** 1 Institute of Hematology and Blood Transfusion, Prague, Czech Republic

## Abstract

**Background:**

Platelets are small anucleated blood particles that play a key role in the control of bleeding. Platelets need to be activated to perform their functions and participate in hemostasis. The process of activation is accompanied by vast protein reorganization and posttranslational modifications. The goal of this study was to identify changes in proteins in platelets activated by different agonists. Platelets were activated by three different agonists - arachidonic acid, collagen, and thrombin. 2D SDS-PAGE (pI 4-7) was used to separate platelet proteins. Proteomes of activated and resting platelets were compared with each other by Progenesis SameSpots statistical software; and proteins were identified by nanoLC-MS/MS.

**Results:**

190 spots were found to be significantly different. Of these, 180 spots were successfully identified and correspond to 144 different proteins. Five proteins were found that had not previously been identified in platelets: protein CDV3 homolog, protein ETHE1, protein LZIC, FGFR1 oncogene partner 2, and guanine nucleotide-binding protein subunit beta-5. Using spot expression profile analysis, we found two proteins (WD repeat-containing protein 1 and mitochondrial glycerol-3-phosphate dehydrogenase) that may be part of thrombin specific activation or signal transduction pathway(s).

**Conclusions:**

Our results, characterizing the differences within proteins in both activated (by various agonists) and resting platelets, can thus contribute to the basic knowledge of platelets and to the understanding of the function and development of new antiplatelet drugs.

## Background

Platelets are small anucleated blood particles derived from megakaryocytes in the bone marrow. They play a key role in the control of bleeding (hemostasis) by the formation of a vascular plug and the release of stimulatory molecules participating in hemostasis. Platelets circulating in the blood are normally maintained in a resting state; but upon a disruption of the integrity of the vascular endothelium or an alternation in the shear stress of the blood flow, platelets become activated. Platelets adhere to the damaged endothelium, coadhere (aggregate), and release both low molecular weight compounds and proteins [1]. This process of activation is accompanied by vast protein reorganization and posttranslational modifications. These platelet proteome changes are not yet fully understood and can lead either to thrombotic or bleeding disorders [1,2]. Therefore, understanding the mechanisms of platelet activation is crucial for the treatment of platelet-involved diseases.

Although the number of identified platelet proteins increases every year, there is still a lack of the supplemental data necessary to clearly establish the role of these proteins in platelet metabolism. Differential proteomics, together with the identification of protein modification by immunoblot or mass spectrometry (MS), are usually used to provide this type of data. In recent years proteomics have become the most popular and broadly used method for studying platelet biology; with several studies exploring both resting and activated platelet proteomes, platelet subproteomes (platelet granules, membrane systems, phosphoproteome, etc.), or studying proteome changes by comparing activated and resting platelets [3-14].

The proteomics methodology enables the exploration of hundreds or even thousands of proteins in a sample using a combination of orthogonal electrophoretic methods, high performance liquid chromatography (HPLC), and MS analyses. Moreover, proteome changes such as posttranslational modifications can be effectively observed only by using proteomic methods. 2D gel electrophoresis has become one of the most widespread proteomic techniques used in proteome analysis (in spite of some disadvantages, such as in the exploration of low-abundance, basic, or low molecular weight proteins). Together with differential image analysis software processing and MS protein identification, these methods are the corner stone of recent proteomic research. Since blood platelets are anucleated particles, the very low levels of retained megakaryocyte-derived mRNA and mitochondrial transcripts hampers genome and transcriptome analyses [15].

The goal of this proteomics study was to identify changes within proteins, in platelets activated by different agonists. Three potent platelet agonists were chosen for platelet activation: arachidonic acid (AA), collagen, and thrombin. All proteomes of either resting or diversely activated platelets were mutually compared using statistical instruments.

## Methods

### Materials

Thrombin, prostaglandin E1, SDS, acrylamide, bis-acrylamide, carrier ampholytes, urea, thiourea, CHAPS, iodoacetamide, TEMED, acetonitrile, methanol (HPLC grade) and all antibodies were purchased from Sigma-Aldrich, Prague (Czech Republic). Immobilized pH gradient (IPG) strips (pH 4-7) and the electrophoresis instruments were acquired from Invitrogen (Carlsbad, CA, USA). Collagen and AA were purchased from Bio/Data (Horsham, PA, USA). Formic acid, DTT, ammonium persulfate, ammonium bicarbonate, and ammonium sulfate were obtained from Fluka Chemie (Buchs, Switzerland). Serva Blue G and Serva Unstained SDS PAGE Protein Marker 6.5 - 200 kDa were acquired from SERVA (Heidelberg, Germany). Sequencing grade, modified trypsin was obtained from Promega (Madison, WI, USA). All other chemicals were purchased from PLIVA-Lachema (Brno, Czech Republic) and were of analytical grade.

### Platelet isolation and activation

Human whole blood was obtained from healthy volunteers who had not been on medication for the previous 14 days. All tested individuals agreed to this study at the time of blood collection. All samples were obtained in accordance with the Ethical Committee regulations of the Institute of Hematology and Blood Transfusion.

Washed blood platelets were isolated by the differential centrifugation of blood collected into a ACD (65 mM citric acid, 85 mM citrate, 111 mM glucose) solution 8.1:1.9 (v/v). Platelet rich plasma (PRP) was obtained by the centrifugation of blood at 250 × g at 37°C for 15 min. PRP with the addition of prostaglandin E1 (1 μM, final concentration) was incubated in a water bath (37°C for 10 min) and centrifuged at 1000 × g at 37°C for 10 min. The platelet pellet was resuspended in a Ca^2+ ^free Tyrode's buffer (140 mM NaCl, 3 mM KCl, 12 mM NaHCO_3_, 0.4 mM NaH_2_PO_4_, 2 mM MgCl_2_, 5.6 mM glucose; pH 6.2) in the presence of 1 μM prostaglandin E1 and centrifuged at 600 × g at 37°C for 10 min. The platelet pellet was then resuspended in a Tyrode's buffer (140 mM NaCl, 3 mM KCl, 12 mM NaHCO_3_, 0.4 mM NaH_2_PO_4_, 1 mM MgCl_2_, 2 mM CaCl_2_, 5.6 mM glucose; pH 7.4) to a final concentration of 5 × 10^8^/mL (standard platelet suspension), and incubated at 37°C for 30 min. The platelet count in PRP was estimated by a Onyx Coulter Counter blood counter (Beckman Coulter, Brea, CA, USA).

Standard platelet suspension (SPS) was divided into four aliquots. Activation of platelets was performed in polypropylene tubes at 37°C while stirring. The described amounts of added agonists were optimized and checked turbidimetrically [16] using a four-channel PAP4 aggregometer (Bio/Data). Tests were performed at 37°C in cuvettes stirred at 1000 rpm. Finally, the optimized amounts of agonists were 10 μL of AA (5 mg/mL) to 500 μL of SPS; 10 μL of collagen (1.9 mg/mL) to 500 μL of SPS; and 10 μL of thrombin (final activity of thrombin 2 NIH U/mL) to 200 μL of SPS. Platelets were stimulated for 10 min (each agonist). The whole platelet suspension aggregated with either agonist was added to four times of the sample volume of cold acetone (-20°C), incubated at -20°C for at least 2 hr, and centrifuged at 15,000 × g at 4°C for 10 min. If not used immediately, the pellets were rapidly frozen and stored at -70°C.

### Two-dimensional gel electrophoresis

Pellets were resuspended in a sample buffer (7 M urea, 2 M thiourea, 4% w/v CHAPS, 65 mM DTT, 1.5% v/v ampholytes, and a trace of bromophenol blue) - 320 μL of sample buffer per pellet, from 1 mL of standard platelet suspension. Samples were then centrifuged at 37,500 × g at 20°C for 1 hr. IPG strips (pI 4-7, 7.7 cm) were rehydrated in 155 μL of the sample for 16 hr at room temperature. The conditions for IEF were: 100 V for 40 min, 200 V for 20 min, 450 V for 15 min, 750 V for 15 min, reaching 2000 V in 10 min, and 4000 Vh (max 0.125 mA and 0.125 W per strip). If not used immediately, strips were rapidly frozen and stored at -70°C. Following IEF, strips were equilibrated for 20 min in a DTT buffer (6 M urea, 50 mM Tris pH 6.8, 2% w/v SDS, 1% w/v DTT, 30% v/v glycerol, and a trace of bromophenol blue), followed by equilibration in an iodoacetamide buffer (6 M urea, 50 mM Tris pH 6.8, 2% w/v SDS, 2.5% w/v iodoacetamide, 30% v/v glycerol, and a trace of bromophenol blue) for another 20 min. Proteins were separated in two dimensions by SDS-PAGE, as described previously [17] (8 × 10 cm, 5-15% gradient gel, 3.75% stacking gel, 5°C, 30 mA/gel) using a P8SD vertical electrophoresis separation system (Owl; Thermo Scientific, Rochester, NY, USA). Following electrophoresis, the gels were stained with colloidal Coomassie blue stain (17% w/v ammonium sulfate, 1.8% H_3_PO_4_, 35% v/v methanol, and 0.12% Serva Blue G) for at least 24 hr and destained with 1% acetic acid.

### Image analysis

Scanned images (16-bit grayscale) were processed and statistically evaluated with Progenesis SameSpots software (Nonlinear Dynamics, Newcastle upon Tyne, UK). Both manual and automatic alignment was used to align the images. All four groups were compared with each other and fold values as well as p-values of all spots were computed by the above mentioned software using one way ANOVA analysis. All spots were prefiltered and manually checked before applying the statistical criteria (Anova p < 0.05 and fold ≥1.2). Normalized spot volumes, instead of spot intensity, were used in statistical processing. Protein identification involved only spots that fulfilled the statistical criteria. Expression profiles (dependence of logarithm of normalized volume on the sample grouping) of all spots, as well as the dendrogram of spot expression profiles, were created automatically by the Progenesis SameSpots software. Because a simple spot could contain more than one protein, the term spot expression profile was used instead of the protein expression profile. The dendrogram is a visual representation of the spot correlation data (with correlation analysis performed on the log normalized spot expression levels). Spots are clustered according their closest correlation. Spots with a high correlation value (close to 1) show similar expression profiles. Experimental pI and Mw values were estimated using the protein Mw marker, together with values of known and identified proteins.

### In-gel digest

Selected spots were excised from the gel and destained in a 1:1 ratio of 100 mM NH_4_HCO_3 _and acetonitrile. Destained gel pieces were dried with acetonitrile, rehydrated with 100 mM NH_4_HCO_3_, and dried with acetonitrile once again. Finally, vacuum dried gel pieces were rehydrated in a trypsin solution (12.5 ng/μL of trypsin in 25 mM NH_4_HCO_3_); and trypsin digestion was performed at 37°C for 16 hr. Then, a 50% acetonitrile/0.1% formic acid solution was added to gel pieces; and peptides were extracted by agitation for 15 min. The supernatant was recovered; and the previous extraction step was repeated. The resulting supernatants were then pooled, dried out, and dissolved in 2% acetonitrile/0.1% formic acid.

### Mass spectrometry analysis

An HCT ultra ion-trap mass spectrometer (Bruker Daltonics, Bremen, Germany) with nanoelectrospray ionization, coupled to a UltiMate 3000 nanoLC system (Dionex, Sunnyvale, CA, USA) was used to perform MS analysis. Tryptic peptides were desalted on a 300 μm ID/5 mm length C18 PepMap 100 precolumn (Dionex) and separated on a 75 μm ID/15 cm length C18 PepMap 100 analytical column (Dionex). A gradient of acetonitrile was used to elute peptides (0% to 20% B for 2 min, 20% to 50% B for 15 min; mobile phase A: 2% ACN/0.1% formic acid, mobile phase B: 80% ACN/0.1% formic acid) at a flow rate of 300 nL/min. The nanoLC system was connected to the MS using a PicoTip needle (New Objective, Woburn, MA, USA) with an applied voltage of 1500 V. The flow of the dry gas was 10 l/min with a dry temperature of 160°C. We used the Standard - Enhanced positive scan mode for CID data acquisition. The scan ranges were 300 - 1500 m/z and 100 - 2500 m/z for MS and MS(2) respectively. Three precursor ions were selected during one autoMS(2) cycle; and active exclusion (0.35 min, singly charged ions) was used. We used esquireControl v6.2 software for data acquisition, DataAnalysis v4.0 for data processing, and BioTools v3.2 (all Bruker Daltonics), together with MASCOT v2.2 (Matrix Science, London, UK) for database searching (SWISS-PROT 57.0, 20403 sequences assigned to Homo sapiens; and NCBInr 20090402, 222100 sequences assigned to Homo sapiens). The following database search parameters were utilized: taxonomy, restricted to Homo sapiens; carbamidomethyl (C), selected as a fixed modification and oxidation (M) as a variable modification; the number of missed cleavages, up to 1; monoisotopic mass; and finally, mass tolerance of 0.1% for MS and 0.5 Da for MS/MS. At least two unique peptides were necessary to identify a protein.

## Results

**Figure 1:**
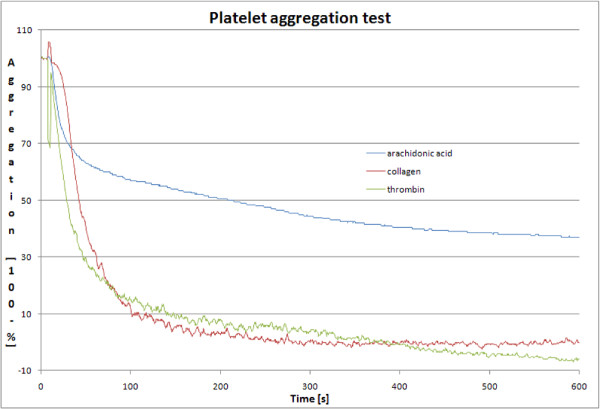
**Aggregation tests**. An example of activation of platelets by the three agonists (arachidonic acid - blue trace, collagen - red trace, and thrombin - green trace). Aggregation tests were performed on each sample of the analysis.

Platelet aggregation tests were performed on each sample to ensure full platelet activation (Figure 1). The amounts of added agonists (as described in Methods) were constant and sufficient to completely activate platelets in all samples with all agonists.

**Figure 2:**
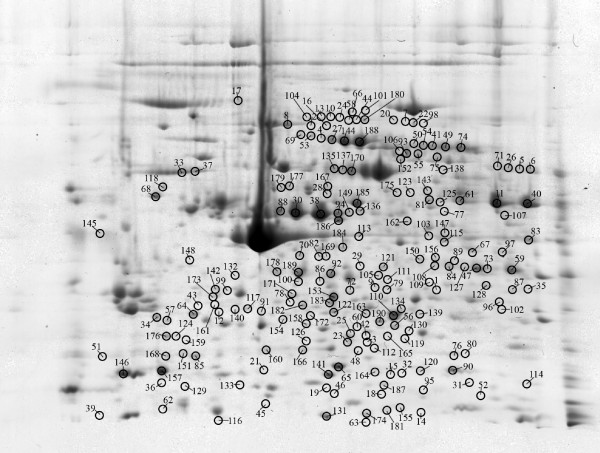
**Positions of significantly differed spots on a 2D gel**. Positions of all spots that were found to significantly differ in 2D gels of resting and activated (by arachidonic acid, collagen, and thrombin) platelets when mutually compared. The 2D gel of resting platelets was used as an illustrative gel to display spot positions.

**Figure 3:**
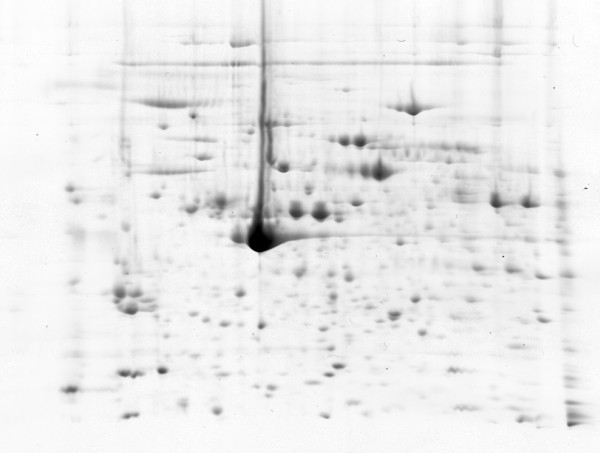
**2D SDS-PAGE of resting platelets**. An illustrative 2D SDS-PAGE gel image of resting platelets without displayed positions of significantly differed spots.

A total of 40 gels were prepared throughout the experiment. Scanned gel images were divided into four individual groups according to platelet activation (resting platelets, AA, collagen, and thrombin activated platelets, respectively). One gel with the sample of collagen-activated platelets was excluded from image analysis due to a pattern distortion. Finally, each group analyzed by the image analysis contained ten gel images, except for the collagen activated platelets, with nine gel images. All four groups were mutually compared using one way ANOVA analysis. Both manual and automatic image alignment were used. Specific pre-filtering and statistical criteria were applied to all spots that were automatically detected by the Progenesis SameSpots software (in total more than 2200 spots detected by the software). Applying pre-filtering (edge and area exclusion, minimal spot area, or normalized volume) altogether more than 1000 spots remained in the analysis. Using statistical criteria in the next step (Anova p < 0.05, minimum fold change 1.2), we detected 190 spots that significantly differed in the normalized volumes. Positions of these spots are presented in an illustrative 2D gel image of resting platelets (Figure 2). The same gel image without spots is presented for a better clarity in Figure 3.

**Figure 4:**
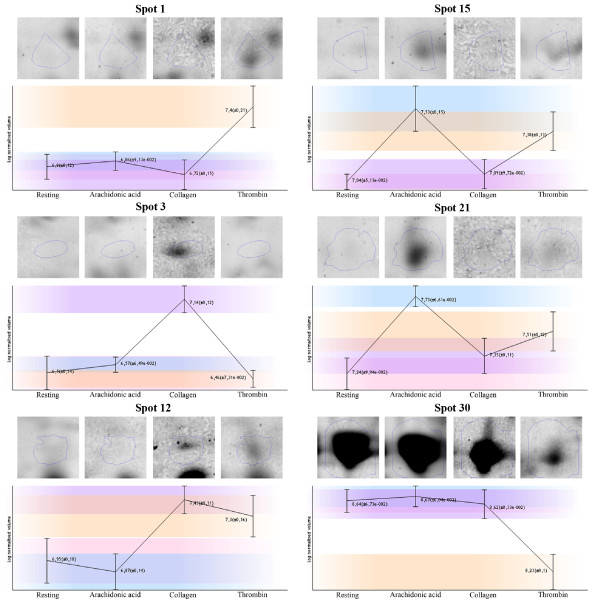
**An example of six detected spots**. Expression profiles with the logarithms of spot expression volumes and their standard deviations for each group (resting platelets and by arachidonic acid, collagen, and thrombin activated platelets as indicated below the x-axis) are presented for six spots. Gel areas containing the same spot from each group (one exemplary gel area from each group) are presented above the logarithm values.

Proteins in 180 of the 190 (95%) spots that differed were successfully identified by nanoLC-MS/MS. At least two unique peptides (fulfilling a minimal Mascot score) were necessary to successfully identify a protein. Actually, three or more unique peptides were found in 91% of proteins. The identified spots correspond to 144 different open reading frames. The list of spots including the fold, Anova p-value, logarithm of normalized volume with its standard deviation, both experimental and calculated values of pI and MW, protein identification with protein accession number, and sequence coverage is summarized in Table 1 (See additional file 1: Table s1). The fold value shows the fold difference between the groups with the lowest and highest normalized volumes. Figure 4 shows an example of six spots presented in Table 1 (See additional file 1: Table s1) with their spot expression profiles and representations of spots in selected areas of illustrative gels from each group.

Whenever the calculated molecular weights of identified proteins using 2D gel analysis were extremely different from the molecular weights based on the LC-MS/MS results with subsequent protein's database search, we distinguished the protein fragments from the full length protein. Fragments of three different proteins were successfully identified (filamin A/FLNA protein, thrombospondin-1/thrombospondin-1 N-terminal domain, and AP-1 complex subunit beta-1/unnamed protein product) by searching NCBInr. They are each represented in the list of spots (See additional file 1: Table s1) in brackets below the original protein identification based on database search. In twelve spots (32, 37, 48, 76, 80, 112, 126, 130, 145, 146, 177, 178) the matching of peptides resulted in an ambiguous identification of proteins (nevertheless all thus identified proteins are represented in the Table 1 and separated by semicolons).

Based on the known properties the identified proteins were categorized into several groups to classify them according to their localization (cytosol, cytoskeleton, Golgi complex, mitochondria, cell membrane, and other). The cytosol group of proteins is prevailing (42.5%). The percent representation of the other localization groups are: cytoskeleton (19%), cell membrane (18%), other (8.5%), Golgi complex (6%) and mitochondria (6%). This representation has to be understood simply as an approximate attribute.

**Figure 5:**
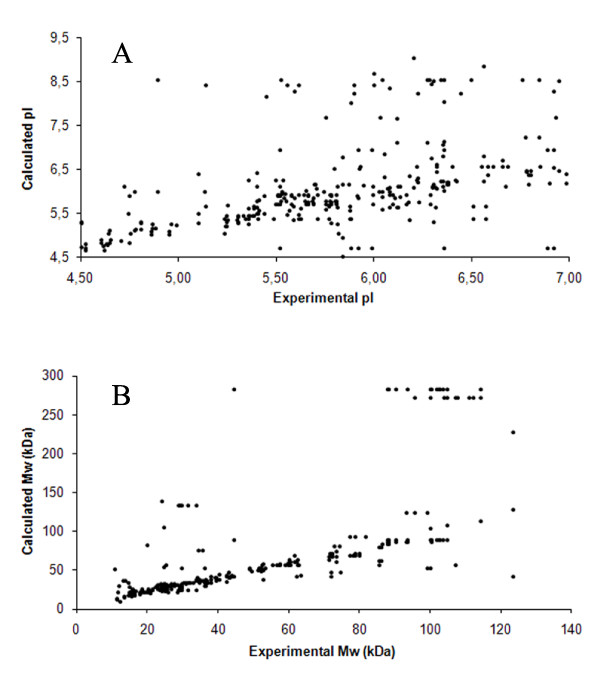
**pI and Mw correlation plots**. A - Correlation between calculated (theoretical) and experimental pI values of all spots; B - Correlation between calculated (theoretical) and experimental Mw values of all spots.

The pI and molecular weight (kDa) of all identified proteins were estimated, as mentioned above. The estimated experimental values were comparatively analyzed against the calculated value of pI and molecular weight of the identified proteins. Both pI and molecular weight correlation plots were created (Figure 5). As expected, comparative analysis of molecular weights showed a stronger correlation (correlation coefficient 0.698) than pI correlation (correlation coefficient 0.488) - this phenomenon is discussed below. More precisely, 71% of the identified proteins showed a difference of not more than 0.5 pI (protein database-based pI values of all proteins in a single spot compared to the spot estimated pI); and 78% of proteins showed a difference less than 15 kDa (in fact, 71% of proteins have a difference less than 8 kDa). Regardless of a strong correlation of protein MW values, large differences between database-based MW and experimental values were found in a few of the identified proteins (thrombospondin-1, talin, filamin-A, multimerin-1, etc.).

Correlation analysis was completed by the Progenesis SameSpots software; and the results were shown in a dendrogram tree (See additional file 2: Figure s6). All spots in the dendrogram were grouped by their spot expression profiles using automatic correlation analysis and hierarchical clustering software. We intentionally chose the top ten spot expression profile groups (with Distance parameter less than 0.5; Distance is equal to 1-C, where C is correlation between spot clusters) with almost identical expression profiles. Thus, each group contains spots with similar expression profiles suggesting that these spots may be co-regulated, co-localized, or by another way co-affected. The list of the expression profile groups; including spot number, experimental and calculated pI and Mw values, the protein identification, and its accession number, is presented in Table 2 (See additional file 1: Table s2). An example of the expression profile group from Table 2 (See additional file 1: Table s2) is presented in Figure 7 (See additional file 3: Figure s7). Spot expression profile group 2 is red colour highlighted in the dendrogram and its expression profile with observed overexpression in AA activated platelets is presented.

All of the identified proteins except the five mentioned below were confirmed to be platelet proteins using the UniProt, PlateletWeb (a database covering the platelet proteome, transcriptome, and interactome, containing information from published platelet proteome and transcriptome studies) [18] and BRENDA databases and literature. We found five proteins that have not been identified in platelets before: protein CDV3 homolog ([Swiss-Prot:Q9UKY7]; spot 72); protein ETHE1, mitochondrial ([Swiss-Prot:O95571]; spot 139); protein LZIC ([Swiss-Prot:Q8WZA0]; spot 151); FGFR1 oncogene partner 2 ([Swiss-Prot:Q9NVK5]; spot 154); and guanine nucleotide-binding protein subunit beta-5 ([Swiss-Prot:O14775]; spot 184).

## Discussion

The aim of this study was to use proteomic tools to explore the human platelet proteome and to extend our knowledge of proteome changes in activated platelets. Isoelectric focusing, mini-gel format SDS-PAGE, and colloidal Coomassie blue staining were used to resolve and visualize proteins, together with nanoLC-MS/MS for protein identification. We used statistical comparative Progenesis SameSpots software to find proteome differences and to perform spot (protein) expression profile analysis. To obtain unique data not yet available we decided to mutually compare resting platelets and platelets activated by AA, collagen, and thrombin. In this paper we present the list of spots with protein identifications that differ among these four mentioned groups. All spots that significantly differed and fulfilled the minimum criteria (p < 0.05, minimum fold change 1.2) were presented in the list of spots (See additional file 1: Table s1). Chosen criteria allow us to compare other sets of results or simply reduce the list of spots by changing the parameters. Cold acetone was used to precipitate both proteins contained in agregates and released by activated platelets in the whole sample. Released proteins thus remained in the sample and therefore we could exclude the effect of the release reaction on our results (e.g. on spot expression).

Despite the limits of the methods used, we have found differences in 190 spots that correspond to 144 unique open reading frames. Five of these proteins have not been identified in platelets before.

Protein CDV3 homolog ([Swiss-Prot:Q9UKY7]; spot 72) is a cytoplasmatic protein of unknown function. It probably plays a role in cell proliferation; and its expression is known to be changed in human breast cancer cells [19]. It was found that this protein is phosphorylated [20,21] and acetylated [22,23]. We found a 2-fold difference in spot normalized volumes when AA activated platelets (and slight difference in thrombin activated platelets) were compared with resting and collagen activated platelets.

Protein ETHE1 ([Swiss-Prot:O95571], ethylmalonic encephalopathy protein 1; spot 139) is probably involved in mitochondria metabolic homeostasis; and it may act as a nuclear-cytoplasmic shuttling protein [24]. ETHE1 overexpression has been observed in hepatoma. It is also known that defects in ETHE1 are a cause of ethylmalonic encephalopathy [25]. Although no enzymatic activity of ETHE1 has yet been determined, sequence similarity shows this protein belongs to the hydrolases. We observed a change of spot normalized volume when AA activated platelets were compared with other groups. The spot 139 contains two proteins, ETHE1 together with F-actin capping protein subunit beta ([Swiss-Prot:P47756]) that is contained in another spots (78, 153, 154, 183). There is a similar spot expression profile shared with spot 78. This indicates the possible postranslational modification of the F-actin capping protein subunit beta, rather than a modification or concentration change of ETHE1.

Protein LZIC ([Swiss-Prot:Q8WZA0], leucine zipper and ICAT homologous domain-containing protein; spot 151) is ubiquitously expressed (the highest level in the kidney); and it seems to be up-regulated in gastric cancers [26]. The zebrafish homolog of human LZIC is required for neuronal survival [27]. We found a difference in spot normalized volumes when AA activated platelets were compared with other groups. A general underexpression is observed in all activated groups when compared to resting platelets.

FGFR1 oncogene partner 2 ([Swiss-Prot:Q9NVK5]; spot 154) is a cytoplasmic protein that may play a role in the wound-healing pathway [28]. There is currently no additional information on its function. This protein is expressed in the bone marrow, spleen, and thymus. Spot volume difference was observed comparing AA and thrombin activated platelets to collagen activated and resting platelets. The second protein identified in the spot (154) is the F-actin capping protein subunit beta ([Swiss-Prot:P47756]). The same spot expression profile as in spot 154 can be observed in another spot (153) containing the F-actin capping protein subunit beta. A similar spot expression profile was found in the spot (183) that contains the F-actin capping protein subunit beta, together with another protein. This situation is identical to that of spot 139 (protein ETHE1, discussed above); therefore, the data do not confirm the modification or concentration change of FGFR1 oncogene partner 2.

Guanine nucleotide-binding protein subunit beta-5 ([Swiss-Prot:O14775]; spot 184) belongs to the G-proteins that are involved in various transmembrane signaling systems [29]. Beta subunits are required for GTPase activity and for G-protein-effector interaction. Similarly to the previously mentioned proteins, we observed a difference in spot normalized volumes comparing AA and thrombin activated platelets to collagen activated and resting platelets. In this spot we have identified a second protein: L-lactate dehydrogenase B chain ([Swiss-Prot:P07195]), which was also found in other spots (82 and 149) that share the same spot expression profile. Assuming this fact, we suppose that the spot volume difference is related to the L-lactate dehydrogenase B chain.

As noted above, spot differences (139, 154, and 184) can be apparently related to co-identified proteins. There are only two proteins (CDV3 and LZIC) from the five new proteins that were uniquely identified within their spots. Both proteins' changes were observed in AA activated platelets. We do not assume that these changes are caused by different protein concentrations, because of platelets' limited proteosynthesis capability [30] and activation conditions (buffered washed isolated platelets activated *ex vivo*without the presence of either other proteins or with traces of plasmatic proteins of the same level in all four sample groups). Therefore, we assume that these changes are caused by posttranslational modifications of CDV3 and LZIC. This assumption is supported by the spot expression profiles of both proteins: their spot normalized volumes are decreased when AA activated platelets are compared to the resting platelet group. The explanation of observing these changes in AA activated platelets may be the involvement of both CDV3 and LZIC proteins in the AA platelet activation pathways via signal transduction or target affecting. A general underexpression of LZIC protein is observed in all activated groups (with strong underexpression in AA activated platelets) when compared to resting platelets. When searched LZIC protein sequence by blast protein tool a putative conserved domain has been detected; LZIC protein seems to belong to beta-catenin-interacting protein superfamily. Beta-catenin is a multifunctional protein involved in cell adhesion, cytoskeleton organization (anchors actin cytoskeleton), signal transduction, protein interactions etc. LZIC protein (zebrafish and mouse) seems to interact with beta-catenin through another protein (or proteins) instead of direct beta-catenin and LZIC interaction [27]. Because platelet activation is accompanied by vast protein and generally cytoskeleton reorganization (including actin cytoskeleton), and LZIC protein regulation (putative underexpression) was observed in all platelet activation groups compared to resting platelet group, we suggest the possible LZIC protein involving in platelet cytoskeleton reorganization during platelet activation process. The mechanism of LZIC protein function in platelet activation may be closely related to AA activation pathways because of stronger underexpression in AA activated platelets and known relation of AA activation pathways to other agonists' pathways. Additional data knowledge (characterization of eventual LZIC protein modification, etc.) should support this assumption.

One of the most important protein posttranslational modifications involved in platelet activation is phosphorylation/dephosphorylation [31,32]. Phosphorylated/dephosphorylated protein spots show a specific pI shift or number of shifts when the protein is multiply-phosphorylated/dephosphorylated (also called spot trains). Spot trains may be caused by modifications other than (de)phosphorylation; and they differ in the pI shift. We did not observe any spot trains of the CDV3 and LZIC spots (72 and 151), so the modification(s) seemed to change substantially and rapidly pIs (not observed in used 4-7 pI range) or molecular weights. This may be caused, among other modifications, by glycosylation. CDV3 and partially LZIC spot normalized volume changes that were observed in AA activated platelets could be caused by other nonspecific protein modifications. Platelet activation by AA (and platelet AA metabolism) is known to generate large amounts of reactive oxygen and nitrogen species (RONS) [33-35]. There are another agonists (thrombin [36], collagen [34,37]) known to produce RONS during platelet activation, but the generation of reactive species is concomitant with AA metabolism [36]. RONS can be generated by NAD(P)H oxidases (NOX), cyclooxygenase 1 (COX-1) and other sources [35]. From the 2D PAGE view, RONS activity can cause not only protein pI shifts, but also can rapidly change protein molecular weights by the crosslinking of proteins. This increase in protein molecular weights can be so large that crosslinked proteins are not able to be detected in the 2D gel (using a gradient gel range of 5-15%). We found several carbonylated and tyrosyl nitrated platelet proteins in our preliminary study when platelets were activated by AA, collagen, and thrombin. Using western blotting we identified in this way posttranslationally modified proteins - for example fibrinogen beta and gamma chains, PDZ and LIM domain protein 1, vinculin, gelsolin, talin-1, moesin and other proteins [38].

Using spot (protein) expression profile analysis, we chose deliberately the top ten discrete spot expression profile groups (SEPG) (See additional file 1: Table s2). This statistical tool contained in Progenesis SameSpots software can be successfully used to extrapolate more information from the measured data and to get a picture of protein relations. It may help to find co-regulated, co-localized, or proteins related by other means. Practically three SEPG (groups 2-3 and 10) contain a simple protein in all their spots in each group (except spot 17 in group 3 and other spot co-identified proteins). Group 2 comprises talin-1; and it seems to be strongly overexpressed in AA activated platelets. Group 3 contains fibrinogen chains (beta and gamma); and it seems to be strongly underexpressed in thrombin activated platelets. This may be caused by an enzymatic activity of thrombin that splits fibrinopeptides A and B from released fibrinogen and yields fibrin monomers polymerizing into a fibrin net. Since platelets contain Factor XIII, catalyzing the formation of covalent bonds between epsilon aminogroups of lysine residues and gamma-carboxyl groups of glutamic acid residues, the fibrin molecules are covalently crosslinked. Fibrin crosslinking greatly affected molecular weights, especially of gamma and alpha fibrinogen chains (gamma-dimers, alpha polymers); and consequently fibrinogen chains levels seem to be underexpressed [39]. Group 10 contains almost only thrombospondin-1 and seems to be strongly overexpressed in thrombin activated platelets in accordance with existing literature. Group 5 could be attached to the previous three groups. More than half of the group 5 spots contain PDZ and LIM domain protein 1; and two of the four remaining spots contain the same pair of proteins (zyxin and serotransferrin). PDZ and LIM domain protein 1 may act as an adapter that brings other proteins to the cytoskeleton. We observed thrombin induced underexpression of this protein in accordance with observed downregulation in TRAP activated platelets [40]. Zyxin is an adhesion plaque protein and a regulator of actin filament assembly. It has been observed that zyxin changes its location (translocation to the platelet surface) in thrombin activated platelets [41] and the protein was found at the peripheral membrane in a differential study of GPVI activated platelets [42]. It has been further shown that zyxin is released from platelets activated by thrombin [43].

There is a category of several SEPG, consisting especially of cytoskeletal proteins (groups 1-2, 8-9). Protein spots of these groups seem to be strongly overexpressed in AA activated platelets (groups 1-2); or they embody a similar spot expression profile (groups 8-9), with putative overexpression in AA and in thrombin activated platelets. The platelet cytoskeleton is rapidly rearranged during platelet activation; and thus cytoskeletal proteins are generally modified to carry out their function. Other factors that can play an important role in protein expression changes are the reactive oxygen and nitrogen species generated during the process of platelet activation, especially in AA activated platelets. The effects of RONS on the modification of proteins were discussed above.

In the three remaining SEPG (4, 6, and 7), group 4 seems to be slightly underexpressed in thrombin activated platelets. This group contains primarily two proteins (pseudo-underexpression of fibrinogen chains was discussed in group 3): WD repeat-containing protein 1 and mitochondrial glycerol-3-phosphate dehydrogenase. WD repeat-containing protein 1 is a cytosolic protein involved in the disassembly of actin filaments. The protein is released by platelets during platelet activation by thrombin [43]. It has been observed that it is translocated to the platelet surface by stimulation with thrombin [41]. Moreover, it has been shown that WD repeat-containing protein 1 interacts with the platelet integrin αIIb regulatory motif [44]. Its possible phosphorylation in thrombin activated platelets has been noted [45]. Mitochondrial glycerol-3-phosphate dehydrogenase (a component of the glycerol phosphate shuttle) is located on the outer surface of the inner mitochondrial membrane. Because of the different localization of both proteins, we can exclude protein modification due to co-localization; and these proteins may be part of the thrombin specific activation or signal transduction pathway(s). Glycerol-3-phosphate dehydrogenase is involved in mitochondria energy production and thus either changes of its presence or its PTM might be expected in all platelet preparations activated independently with the three mentioned agonists. Surprisingly, only in thrombin activated platelets both the spots 26 and 71, containing glycerol-3-phosphate dehydrogenase, show strong underexpression (2.8 and 2.0 fold change) and no change in their expression was observed in AA or collagen activated platelets and in resting platelets. We have not yet obtained any information about possible PTMs of these proteins that could induce underexpression. Nevertheless, the phosphorylation of WD repeat-containing protein 1 and oxidative modifications of both proteins might be probably present. Due to different localizations of both proteins in platelets we speculate that these SEPG 4 proteins can mutually interact via another protein or proteins. The absence of such interaction-mediator(s) in SEPG 4 can be explained by the limits of 2D electrophoresis (detection and pI range limits etc.).

Group 6 contains several cytoplasmatic and cytoskeletal proteins. The majority of them are components of the cytoskeleton or are involved in cytoskeleton reorganization and interaction. This group of spots differs slightly in AA activated platelets and could be classified with the other spot groups containing cytoplasmatic proteins changed in AA activated platelets (groups 1-2, 8-9). Group 7 contains proteins that were changed in activated platelets, no matter which agonist was used for activation. This probably indicates that all of these proteins may be involved in platelet activation pathways or other differing functions that generally take place in activated platelets. For example, mitochondrial thioredoxin-dependent peroxide reductase is an enzyme that is involved in the redox regulation of the cell and protects other enzymes from oxidative damage (which is increased during platelet activation) [46].

Many of proteins identified in this study have been found to change their expression agonist-dependently. As an example, thrombospondin-1 which is changed in thrombin activated platelets (spots 1, 9, 79 etc.) is an adhesive glycoprotein and is involved in platelet aggregation. Glutathione peroxidase 1 changed in collagen activated platelets (spots 3, 23, 25 and 42) protects from oxidative stress. Filamin-A changed in AA activated platelets (spots 7, 20, 22 and 98) is involved in actin cytoskeleton reorganization. Talin-1 changed in AA activated platelets (spots 10, 24, 58 etc.) is involved in cytoskeleton reorganization.

In spite of repeated MS/MS analysis, ten spots of detected 190 spots, that significantly differed in the normalized volumes, did not yield any valid spectra. Therefore, the proteins present in these spots remained unidentified. All of these spots had relatively low molecular weight (the most of them with MW lower than 13 kDa), thus the amount of tryptic peptides might not be sufficient for the identification. These peptides could also be lost in the pre-concentration step during nanoLC-MS/MS analysis, due to their low affinity with the C18 TRAP column. Moreover, these proteins can be proteoglycans yielding fewer tryptic peptides. The profile analysis of these spots expression (data not shown) indicates a group of five spots that could contain the same protein or a group of the same proteins. These spots (19, 39, 45, 46, and 51) represent the same expression profile; and their molecular weights are similar.

The comparative analysis of protein molecular weights showed a stronger correlation than the pI analysis. This indicates that most protein changes can be assigned to posttranslational modifications (phosphorylation, oxidation, etc.). These modifications only slightly change protein molecular weights in 2D PAGE maps, but can notably affect the pI of proteins. By contrast, we observed several proteins showing a significant difference in estimated and calculated molecular weights (thrombospondin-1, talin, filamin-A, etc.). However, no identification of intact proteins (of the calculated molecular weight) corresponding to those fragments was observed. Similar results have been already reported by other authors [47].

## Conclusions

In conclusion, the reported data show substantial changes in proteomes of platelets activated by various agonists. We found five proteins that have not been identified in platelets before. Several proteins have been found to change agonist dependently. Two candidate thrombin pathway specific proteins have been identified: WD repeat-containing protein 1 and mitochondrial glycerol-3-phosphate dehydrogenase. Nevertheless, further investigation has to be done to clarify their meaning in the thrombin activation pathway. Our results, characterizing the differences in proteins in activated and resting platelets, contribute to the basic knowledge of platelets and to development of new antiplatelet drugs.

## Competing interests

The authors declare that they have no competing interests.

## Authors' contributions

PM and ZR designed and performed research, analyzed data and wrote the manuscript. JŠ and AS performed research. JS and JED designed research and wrote the manuscript. All authors read and approved the final manuscript.

## Supplementary Material

Additional file 1 **Additional Tables 1 and 2**. Table 1 - List of spots that significantly differ in activated platelets. Table 2 - Top ten spot expression profile groups. Click here for file

Additional file 2 **Figure 6 - The dendrogram tree**. The dendrogram tree - a visual representation of the spot correlation data of all spots that were found to be significantly different among all four platelet groups (resting and activated platelets) when mutually compared. Click here for file

Additional file 3 **Figure 7 - An example of the expression profile group (group 2)**. A - The dendrogram tree with highlighted (in red colour) spots of spot expression profile group 2. The x-axis is composed of all 190 spots that significantly differed when resting and by different agonist activated platelet proteomes were mutually compared. Spots are grouped by their expression patterns using correlation analysis and hierarchical clustering.; B - The expression profile of spot expression profile group 2 spots. All spots for each activation group (resting platelets and by arachidonic acid, collagen, and thrombin activated platelets as indicated below the x-axis) are displayed. Click here for file
