# Supplementary material for: Proteome changes in platelets activated by arachidonic acid, collagen, and thrombin
Source: Proteome Sci. 2010 Nov 12;8:56. doi: 10.1186/1477-5956-8-56 (PMC2996359; doi:10.1186/1477-5956-8-56)
Supplement: Additional file 1 — Additional Tables 1 and 2. Table 1 - List of spots that significantly differ in activated platelets. Table 2 - Top ten spot expression profile groups. [file 1477-5956-8-56-S1.PDF]

Table I. List of spots that significantly differ in activated platelets.

			Log(V_N)				Experimental		Calculated				
Spot	Fold	Anova (*p*)	N	A	C	T	pI	Mw (kDa)	pI	Mw (kDa)	Protein identification	AN	SC (%)
1	5.1	3.143E-11	6.80 ± 0.12	6.86 ± 0.09	6.72 ± 0.14	7.40 ± 0.21	6.36	29	4.71	133	Thrombospondin-1	P07996	10
									6.95	24	[Thrombospondin-1 N-Terminal Domain]	gi|88191913	65
2	5.0	2.783E-10	6.94 ± 0.13	7.34 ± 0.15	6.88 ± 0.20	7.58 ± 0.20	5.41	99	5.37	52	Fibrinogen gamma chain	P02679	44
									5.50	124	Vinculin	P18206	8
3	4.9	9.992E-16	6.50 ± 0.14	6.57 ± 0.06	7.14 ± 0.11	6.46 ± 0.07	5.84	20	6.15	22	Glutathione peroxidase 1	P07203	49
4	4.4	2.958E-13	7.16 ± 0.11	7.80 ± 0.11	7.21 ± 0.13	7.52 ± 0.13	5.52	94	5.70	283	Filamin-A	P21333	12
									5.93	89	[FLNA protein]	gi|15779184	39
									5.90	86	Gelsolin	P06396	11
5	4.2	3.000E-08	7.68 ± 0.16	7.72 ± 0.19	7.62 ± 0.23	7.11 ± 0.13	6.90	72	6.17	67	WD repeat-containing protein 1	O75083	36
6	4.0	1.083E-07	7.84 ± 0.21	7.90 ± 0.17	7.81 ± 0.19	7.28 ± 0.19	6.99	71	6.17	67	WD repeat-containing protein 1	O75083	21
									6.40	63	Stress-induced-phosphoprotein 1	P31948	13
7	4.0	2.624E-07	6.94 ± 0.19	7.55 ± 0.21	7.11 ± 0.21	7.02 ± 0.13	6.10	103	5.70	283	Filamin-A	P21333	14
									5.93	89	[FLNA protein]	gi|15779184	46
8	3.9	4.640E-11	7.88 ± 0.14	8.08 ± 0.12	7.86 ± 0.10	8.44 ± 0.15	5.36	100	5.25	104	Alpha-actinin-1	P12814	23
									5.37	52	Fibrinogen gamma chain	P02679	42
9	3.8	5.612E-13	7.20 ± 0.11	7.19 ± 0.05	7.22 ± 0.07	7.75 ± 0.17	5.88	30	4.71	133	Thrombospondin-1	P07996	8
									8.00	28	[Thrombospondin-1 N-Terminal Domain]	gi|88191917	39
									5.37	52	Fibrinogen gamma chain	P02679	24
10	3.8	6.898E-11	7.04 ± 0.10	7.59 ± 0.16	7.06 ± 0.10	7.11 ± 0.15	5.55	107	5.77	272	Talin-1	Q9Y490	17
11	3.6	1.554E-15	8.79 ± 0.07	8.72 ± 0.08	8.75 ± 0.09	8.23 ± 0.11	6.76	53	8.54	57	Fibrinogen beta chain	P02675	53
12	3.5	1.726E-09	6.95 ± 0.18	6.87 ± 0.14	7.43 ± 0.10	7.30 ± 0.16	4.90	25	8.54	57	Fibrinogen beta chain	P02675	42
									5.98	23	Heat shock protein beta-1	P04792	40
13	3.5	6.496E-09	7.32 ± 0.07	7.83 ± 0.18	7.35 ± 0.10	7.36 ± 0.19	5.52	107	5.77	272	Talin-1	Q9Y490	17
									8.54	57	Fibrinogen beta chain	P02675	13
14	3.3	2.043E-07	6.83 ± 0.12	7.33 ± 0.17	6.85 ± 0.15	7.00 ± 0.18	6.20	11	9.04	14	Platelet basic protein	P02775	37
15	3.3	5.276E-11	7.04 ± 0.05	7.53 ± 0.15	7.09 ± 0.09	7.38 ± 0.13	6.00	16	8.67	25	Vesicle-trafficking protein SEC22b	O75396	15
									8.41	23	Transgelin-2	P37802	20
16	3.2	1.068E-07	6.84 ± 0.15	7.15 ± 0.16	6.88 ± 0.11	7.33 ± 0.19	5.51	100	5.70	283	Filamin-A	P21333	7
									5.93	89	[FLNA protein]	gi|15779184	24
									5.90	86	Gelsolin	P06396	9
17	3.2	2.678E-07	8.06 ± 0.20	8.36 ± 0.14	7.87 ± 0.09	8.09 ± 0.09	5.10	124	5.50	228	Myosin-9	P35579	18
									5.29	42	Actin, cytoplasmic 1	P60709	30
									6.39	128	Integrin alpha-6	P23229	6
18	3.1	1.466E-08	6.57 ± 0.21	7.05 ± 0.10	6.54 ± 0.15	6.96 ± 0.16	5.93	14	6.56	37	PDZ and LIM domain protein 1	O00151	6
19	3.1	2.142E-08	6.43 ± 0.15	6.92 ± 0.14	6.46 ± 0.15	6.82 ± 0.18	5.53	14			unidentified		
20	3.1	3.456E-06	7.06 ± 0.16	7.56 ± 0.17	7.20 ± 0.19	7.12 ± 0.19	6.00	102	5.70	283	Filamin-A	P21333	13
									5.93	89	[FLNA protein]	gi|15779184	42
21	3.1	1.963E-11	7.24 ± 0.10	7.73 ± 0.07	7.35 ± 0.11	7.51 ± 0.12	5.14	16	8.41	23	Transgelin-2	P37802	17

22	3.0	7.647E-06	7.13 ± 0.19	7.60 ± 0.23	7.24 ± 0.14	7.20 ± 0.12	6.17	103	5.70	283	Filamin-A	P21333	15
									5.93	89	[FLNA protein]	gi|15779184	47
23	3.0	2.153E-10	7.39 ± 0.08	7.43 ± 0.14	6.95 ± 0.12	7.38 ± 0.10	5.70	20	6.15	22	Glutathione peroxidase 1	P07203	41
24	2.9	4.718E-09	7.01 ± 0.14	7.43 ± 0.14	7.03 ± 0.11	6.97 ± 0.11	5.65	111	5.77	272	Talin-1	Q9Y490	15
25	2.9	1.056E-05	7.07 ± 0.20	7.17 ± 0.14	6.72 ± 0.07	7.10 ± 0.19	5.70	21	6.15	22	Glutathione peroxidase 1	P07203	28
26	2.8	5.755E-07	7.64 ± 0.14	7.67 ± 0.16	7.64 ± 0.17	7.22 ± 0.16	6.84	73	7.23	81	Glycerol-3-phosphate dehydrogenase, mitochondrial	P43304	11
27	2.8	2.234E-13	7.78 ± 0.06	8.23 ± 0.09	7.78 ± 0.07	7.98 ± 0.11	5.58	90	5.70	283	Filamin-A	P21333	12
									5.93	89	[FLNA protein]	gi|15779184	38
									5.90	86	Gelsolin	P06396	18
28	2.8	5.944E-10	6.74 ± 0.13	7.19 ± 0.10	6.79 ± 0.12	6.92 ± 0.08	5.54	60	5.95	63	Dihydropyrimidinase-related protein 2	Q16555	21
29	2.7	4.292E-09	7.21 ± 0.12	7.59 ± 0.17	7.19 ± 0.10	7.61 ± 0.14	5.80	36	6.52	76	Fermitin family homolog 3	Q86UX7	14
30	2.7	3.797E-14	8.64 ± 0.07	8.67 ± 0.06	8.62 ± 0.08	8.23 ± 0.10	5.35	52	5.37	52	Fibrinogen gamma chain	P02679	41
31	2.7	2.160E-05	6.79 ± 0.13	7.19 ± 0.22	6.86 ± 0.09	7.01 ± 0.15	6.56	15	8.85	22	Peroxiredoxin-5, mitochondrial	P30044	47
32	2.7	6.371E-11	7.11 ± 0.10	7.54 ± 0.12	7.17 ± 0.08	7.51 ± 0.12	6.06	16	6.32; 6.84	21; 21	ADP-ribosylation factor 1; ADP-ribosylation factor 3	P84077; P61204	19; 19
33	2.7	4.906E-04	7.85 ± 0.14	7.48 ± 0.21	7.89 ± 0.23	7.83 ± 0.24	4.78	75	5.14	47	Serum deprivation-response protein	O95810	21
34	2.6	2.100E-02	7.38 ± 0.07	7.13 ± 0.14	7.38 ± 0.34	7.25 ± 0.09	4.61	25	4.89	24	Synaptosomal-associated protein 23	O00161	35
									4.84	26	Ubiquitin carboxyl-terminal hydrolase isozyme L3	P15374	22
35	2.6	1.041E-07	7.35 ± 0.07	7.61 ± 0.11	7.31 ± 0.18	7.73 ± 0.15	6.92	30	8.27	52	Adenylyl cyclase-associated protein 1	Q01518	10
									4.71	133	Thrombospondin-1	P07996	5
									6.95	24	[Thrombospondin-1 N-Terminal Domain]	gi|88191913	27
									6.54	32	S-formylglutathione hydrolase	P10768	26
36	2.6	4.402E-06	6.84 ± 0.15	7.25 ± 0.14	6.80 ± 0.21	7.09 ± 0.17	4.64	15	5.05	28	Calpain small subunit 1	P04632	13
37	2.6	2.000E-03	7.32 ± 0.15	6.97 ± 0.25	7.34 ± 0.22	7.38 ± 0.27	4.81	72	5.14	47	Serum deprivation-response protein	O95810	21
									5.29; 5.31	42; 42	Actin, cytoplasmic 1; Actin, cytoplasmic 2	P60709; P63261	17; 17
38	2.5	8.944E-13	8.74 ± 0.08	8.73 ± 0.07	8.72 ± 0.07	8.32 ± 0.11	5.49	52	5.37	52	Fibrinogen gamma chain	P02679	48
39	2.5	3.388E-04	6.48 ± 0.17	6.85 ± 0.22	6.53 ± 0.15	6.71 ± 0.18	4.49	12			unidentified		
40	2.5	3.206E-07	8.79 ± 0.14	8.79 ± 0.12	8.74 ± 0.13	8.39 ± 0.15	6.85	57	8.54	57	Fibrinogen beta chain	P02675	48
41	2.5	5.295E-07	7.90 ± 0.06	7.50 ± 0.09	7.86 ± 0.19	7.81 ± 0.15	6.34	86	6.22	62	Zyxin	Q15942	30
									8.54	57	Fibrinogen beta chain	P02675	19
42	2.5	2.611E-04	6.57 ± 0.13	6.67 ± 0.16	6.93 ± 0.20	6.53 ± 0.22	5.87	21	6.15	22	Glutathione peroxidase 1	P07203	33
43	2.4	7.781E-07	6.94 ± 0.20	6.86 ± 0.17	7.28 ± 0.08	7.23 ± 0.10	4.78	25	5.98	23	Heat shock protein beta-1	P04792	35
44	2.4	2.238E-07	7.18 ± 0.13	7.54 ± 0.13	7.16 ± 0.10	7.25 ± 0.12	5.77	100	5.77	272	Talin-1	Q9Y490	15
									5.70	283	Filamin-A	P21333	5
									5.93	89	[FLNA protein]	gi|15779184	18
45	2.4	4.200E-07	6.56 ± 0.16	6.86 ± 0.11	6.61 ± 0.12	6.95 ± 0.14	5.13	13			unidentified		
46	2.4	1.894E-05	6.88 ± 0.20	7.16 ± 0.10	6.95 ± 0.18	7.27 ± 0.14	5.61	14			unidentified		
47	2.4	1.177E-07	8.15 ± 0.11	7.78 ± 0.16	8.17 ± 0.11	8.09 ± 0.10	6.61	35	6.56	37	PDZ and LIM domain protein 1	O00151	49
48	2.4	5.604E-05	6.18 ± 0.12	6.47 ± 0.22	6.22 ± 0.08	6.53 ± 0.18	5.81	19	5.64; 6.12	25; 24	Ras-related protein Rab-11B; Ras-related protein Rab-11A	Q15907; P62491	21; 21
49	2.3	7.983E-09	8.07 ± 0.07	7.70 ± 0.08	7.97 ± 0.15	7.82 ± 0.07	6.35	86	6.22	62	Zyxin	Q15942	15
									6.81	79	Serotransferrin	P02787	5

50	2.3	2.452E-06	7.58 ± 0.09	7.28 ± 0.13	7.64 ± 0.17	7.64 ± 0.14	6.18	88	5.75	84	Coagulation factor XIII A chain	P00488	15
51	2.3	1.973E-04	6.65 ± 0.18	6.78 ± 0.08	6.48 ± 0.13	6.83 ± 0.19	4.50	18			unidentified		
52	2.3	3.098E-04	6.67 ± 0.18	7.01 ± 0.19	6.64 ± 0.16	6.65 ± 0.20	6.69	13	6.56	37	PDZ and LIM domain protein 1	O00151	16
53	2.3	7.859E-08	7.39 ± 0.08	7.59 ± 0.12	7.33 ± 0.06	7.68 ± 0.15	5.44	93	5.50	124	Vinculin	P18206	14
									5.90	86	Gelsolin	P06396	18
54	2.3	3.095E-08	7.81 ± 0.07	7.45 ± 0.11	7.80 ± 0.15	7.75 ± 0.10	6.23	87	5.75	84	Coagulation factor XIII A chain	P00488	14
55	2.3	7.667E-06	7.66 ± 0.10	7.29 ± 0.14	7.53 ± 0.12	7.43 ± 0.15	6.20	78	6.08	68	Moesin	P26038	16
56	2.2	5.576E-10	7.27 ± 0.10	6.93 ± 0.09	7.18 ± 0.09	6.92 ± 0.09	6.03	23	7.67	28	Thioredoxin-dependent peroxide reductase, mitochondrial	P30048	24
57	2.2	4.602E-05	7.10 ± 0.04	6.72 ± 0.18	7.01 ± 0.22	6.96 ± 0.10	4.65	23	4.89	24	Synaptosomal-associated protein 23	O00161	43
58	2.2	6.796E-05	7.02 ± 0.15	7.36 ± 0.14	7.03 ± 0.16	7.11 ± 0.16	5.68	108	5.77	272	Talin-1	Q9Y490	14
59	2.1	2.916E-06	8.44 ± 0.11	8.10 ± 0.16	8.42 ± 0.12	8.35 ± 0.09	6.85	34	6.56	37	PDZ and LIM domain protein 1	O00151	41
60	2.1	6.289E-10	6.98 ± 0.09	7.23 ± 0.06	7.20 ± 0.08	7.31 ± 0.08	5.75	23	7.67	28	Thioredoxin-dependent peroxide reductase, mitochondrial	P30048	24
61	2.1	7.467E-08	8.32 ± 0.07	8.27 ± 0.09	8.27 ± 0.10	7.97 ± 0.14	6.50	58	8.54	57	Fibrinogen beta chain	P02675	44
62	2.1	3.979E-06	7.33 ± 0.08	7.60 ± 0.14	7.41 ± 0.13	7.65 ± 0.12	4.62	12	4.67	29	Tropomyosin alpha-4 chain	P67936	14
63	2.1	1.900E-02	5.73 ± 0.16	6.05 ± 0.19	5.92 ± 0.21	5.97 ± 0.26	5.82	11	5.05	51	Tubulin beta-1 chain	Q9H4B7	15
64	2.1	1.000E-03	7.65 ± 0.09	7.31 ± 0.16	7.59 ± 0.26	7.55 ± 0.16	4.76	24	5.05	28	Calpain small subunit 1	P04632	24
65	2.1	7.385E-12	8.20 ± 0.06	8.52 ± 0.09	8.23 ± 0.05	8.35 ± 0.05	5.62	17	8.41	23	Transgelin-2	P37802	29
66	2.1	1.020E-05	7.20 ± 0.10	7.47 ± 0.12	7.17 ± 0.12	7.14 ± 0.16	5.76	114	5.77	272	Talin-1	Q9Y490	17
									5.70	283	Filamin-A	P21333	4
									5.58	113	Ubiquitin-like modifier-activating enzyme 7	P41226	4
67	2.0	1.453E-05	7.38 ± 0.12	7.08 ± 0.14	7.40 ± 0.11	7.29 ± 0.12	6.59	40	6.37	40	Twinfilin-2	Q6IBS0	24
									6.56	37	PDZ and LIM domain protein 1	O00151	20
68	2.0	7.000E-03	7.81 ± 0.17	7.49 ± 0.20	7.74 ± 0.16	7.70 ± 0.22	4.61	59	4.76	57	Protein disulfide-isomerase	P07237	25
69	2.0	4.000E-03	7.24 ± 0.10	7.36 ± 0.10	7.19 ± 0.05	7.43 ± 0.23	5.40	96	5.77	272	Talin-1	Q9Y490	6
									5.50	124	Vinculin	P18206	11
70	2.0	7.000E-03	7.32 ± 0.18	7.25 ± 0.22	7.14 ± 0.18	6.99 ± 0.20	5.36	38	6.26	42	Beta-parvin	Q9HBI1	36
									5.45	33	F-actin capping protein subunit alpha-1	P52907	34
									5.60	38	Guanine nucleotide-binding protein G(I)/G(S)/G(T) subunit beta-1	P62873	9
71	2.0	5.562E-05	7.49 ± 0.13	7.56 ± 0.11	7.46 ± 0.15	7.26 ± 0.10	6.78	74	7.23	81	Glycerol-3-phosphate dehydrogenase, mitochondrial	P43304	16
72	2.0	2.000E-03	6.41 ± 0.20	6.15 ± 0.06	6.41 ± 0.14	6.27 ± 0.17	5.71	29	6.06	27	Protein CDV3 homolog	Q9UKY7	40
73	2.0	1.299E-05	7.83 ± 0.11	7.57 ± 0.09	7.86 ± 0.15	7.78 ± 0.07	6.66	34	6.70	40	Aflatoxin B1 aldehyde reductase member 2	O43488	12
									6.56	37	PDZ and LIM domain protein 1	O00151	42
74	2.0	8.670E-05	8.24 ± 0.11	7.93 ± 0.14	8.11 ± 0.16	7.98 ± 0.13	6.56	86	6.81	79	Serotransferrin	P02787	14
									6.22	62	Zyxin	Q15942	23
75	2.0	3.720E-04	7.55 ± 0.14	7.28 ± 0.12	7.44 ± 0.14	7.22 ± 0.22	6.32	77	6.08	68	Moesin	P26038	12
									5.63	93	Caldesmon	Q05682	8
76	2.0	5.000E-03	7.43 ± 0.10	7.62 ± 0.18	7.50 ± 0.21	7.34 ± 0.11	6.51	18	5.65; 5.37	21; 21	Ras-related protein Rap-1b; Ras-related protein Rap-1b-like protein	P61224; A6NIZ1	35; 35
77	2.0	1.507E-04	6.06 ± 0.19	6.15 ± 0.10	6.39 ± 0.08	6.15 ± 0.14	6.42	53	6.24	50	Septin-6	Q14141	18
78	2.0	1.879E-04	5.90 ± 0.20	6.05 ± 0.10	5.93 ± 0.16	6.23 ± 0.12	5.30	27	5.36	32	F-actin capping protein subunit beta	P47756	13
79	2.0	3.280E-04	6.36 ± 0.13	6.30 ± 0.13	6.32 ± 0.12	6.58 ± 0.16	5.99	29	4.71	133	Thrombospondin-1	P07996	5
									6.95	24	[Thrombospondin-1 N-Terminal Domain]	gi|88191913	32

80	2.0	4.300E-02	7.40 ± 0.14	7.58 ± 0.22	7.43 ± 0.24	7.31 ± 0.19	6.57	20	5.65; 5.37	21; 21	Ras-related protein Rap-1b; Ras-related protein Rap-1b-like protein	P61224; A6NIZ1	28; 28
81	1.9	1.420E-04	7.26 ± 0.12	7.32 ± 0.15	7.27 ± 0.09	7.03 ± 0.14	6.28	57	8.54	57	Fibrinogen beta chain	P02675	35
82	1.9	9.313E-04	6.68 ± 0.10	6.47 ± 0.20	6.59 ± 0.11	6.39 ± 0.14	5.50	38	6.26	42	Beta-parvin	Q9HBI1	14
									5.71	37	L-lactate dehydrogenase B chain	P07195	15
83	1.9	6.000E-03	7.94 ± 0.13	7.79 ± 0.15	7.67 ± 0.12	7.76 ± 0.19	6.95	43	8.50	40	Pleckstrin	P08567	15
									6.46	47	Fumarylacetoacetase	P16930	17
84	1.9	1.393E-06	7.55 ± 0.10	7.31 ± 0.08	7.58 ± 0.10	7.46 ± 0.09	6.40	36	6.56	37	PDZ and LIM domain protein 1	O00151	37
85	1.9	3.476E-05	7.71 ± 0.08	7.54 ± 0.09	7.52 ± 0.14	7.43 ± 0.10	4.78	18	5.12	19	Galectin-related protein	Q3ZCW2	27
86	1.9	8.814E-05	6.82 ± 0.12	6.88 ± 0.09	6.86 ± 0.13	7.09 ± 0.12	5.52	32	4.71	133	Thrombospondin-1	P07996	12
									6.95	24	[Thrombospondin-1 N-Terminal Domain]	gi|88191913	65
									6.11	34	Syntaxin-11	O75558	24
87	1.9	3.800E-02	7.25 ± 0.14	7.49 ± 0.21	7.36 ± 0.16	7.39 ± 0.15	6.89	29	4.71	133	Thrombospondin-1	P07996	3
									6.95	24	[Thrombospondin-1 N-Terminal Domain]	gi|88191913	16
88	1.8	3.688E-07	8.01 ± 0.10	8.06 ± 0.05	7.98 ± 0.05	7.78 ± 0.12	5.24	52	5.37	52	Fibrinogen gamma chain	P02679	32
									5.05	51	Tubulin beta-1 chain	Q9H4B7	22
89	1.8	1.153E-05	6.75 ± 0.10	6.50 ± 0.13	6.77 ± 0.10	6.60 ± 0.10	6.42	37	6.26	34	Crk-like protein	P46109	12
90	1.8	4.336E-07	8.08 ± 0.09	8.04 ± 0.07	7.86 ± 0.07	8.12 ± 0.08	6.44	16	8.22	19	Cofilin-1	P23528	48
91	1.8	3.849E-05	7.44 ± 0.08	7.54 ± 0.12	7.69 ± 0.11	7.63 ± 0.09	5.14	25	5.98	23	Heat shock protein beta-1	P04792	20
92	1.8	6.119E-09	7.71 ± 0.05	7.47 ± 0.08	7.73 ± 0.05	7.67 ± 0.09	5.57	33	5.57	33	F-actin-capping protein subunit alpha-2	P47755	46
93	1.8	1.200E-02	7.35 ± 0.13	7.09 ± 0.13	7.17 ± 0.20	7.09 ± 0.24	6.12	82	5.63	93	Caldesmon	Q05682	10
94	1.8	1.577E-07	7.85 ± 0.09	7.84 ± 0.08	7.88 ± 0.05	7.62 ± 0.09	5.63	52	5.61	48	Actin-related protein 3	P61158	26
									5.37	52	Fibrinogen gamma chain	P02679	13
95	1.8	1.733E-04	7.17 ± 0.07	7.39 ± 0.15	7.14 ± 0.12	7.29 ± 0.11	6.30	14	6.75	16	Hemoglobin subunit beta	P68871	48
									8.44	15	Profilin-1	P07737	40
96	1.8	1.031E-04	6.83 ± 0.05	7.03 ± 0.11	7.07 ± 0.11	6.97 ± 0.12	6.78	27	6.46	25	26S proteasome non-ATPase regulatory subunit 9	O00233	21
									6.45	32	Purine nucleoside phosphorylase	P00491	21
97	1.8	8.980E-06	7.06 ± 0.09	6.88 ± 0.09	7.13 ± 0.10	7.07 ± 0.09	6.79	39	6.37	40	Twinfilin-2	Q6IBS0	35
									6.16	40	Mannose-1-phosphate guanyltransferase beta	Q9Y5P6	14
98	1.8	8.000E-03	6.97 ± 0.09	7.17 ± 0.20	7.02 ± 0.09	6.94 ± 0.15	6.29	104	5.70	283	Filamin-A	P21333	14
									5.93	89	[FLNA protein]	gi|15779184	47
99	1.8	8.000E-03	6.87 ± 0.12	6.66 ± 0.12	6.90 ± 0.16	6.78 ± 0.20	4.96	30	5.02	30	Microtubule-associated protein RP/EB family member 1	Q15691	42
									5.09	27	Chloride intracellular channel protein 1	O00299	12
100	1.8	3.000E-03	7.18 ± 0.13	6.98 ± 0.16	7.24 ± 0.13	7.20 ± 0.16	5.36	32	5.45	33	F-actin-capping protein subunit alpha-1	P52907	15
101	1.7	7.000E-03	7.15 ± 0.13	7.37 ± 0.15	7.10 ± 0.22	7.12 ± 0.18	5.80	112	5.77	272	Talin-1	Q9Y490	13
102	1.7	4.000E-02	5.92 ± 0.13	6.12 ± 0.21	5.94 ± 0.20	6.13 ± 0.21	6.81	26	6.46	25	26S proteasome non-ATPase regulatory subunit 9	O00233	21

103	1.7	7.000E-03	7.08 ± 0.20	6.85 ± 0.14	7.09 ± 0.14	7.07 ± 0.13	6.31	43	6.15	42	Septin-2	Q15019	14
									5.31	42	Actin, cytoplasmic 2	P63261	21
									8.50	40	Pleckstrin	P08567	12
104	1.7	2.400E-02	7.02 ± 0.09	7.23 ± 0.18	7.02 ± 0.09	7.17 ± 0.25	5.40	104	5.77	272	Talin-1	Q9Y490	11
105	1.7	6.205E-04	6.55 ± 0.11	6.70 ± 0.09	6.63 ± 0.14	6.79 ± 0.11	5.92	34	5.84	38	Serine/threonine-protein phosphatase PP1-beta catalytic subunit	P62140	17
									4.71	133	Thrombospondin-1	P07996	3
									6.95	24	[Thrombospondin-1 N-Terminal Domain]	gi|88191913	21
106	1.7	2.000E-03	6.59 ± 0.12	6.34 ± 0.17	6.55 ± 0.06	6.49 ± 0.13	6.08	80	6.08	68	Moesin	P26038	7
									5.92	71	Serum albumin	P02768	10
107	1.7	3.000E-03	7.09 ± 0.11	6.85 ± 0.12	6.99 ± 0.18	6.85 ± 0.17	6.80	51	6.36	50	Septin-11	Q9NVA2	12
108	1.7	7.653E-07	7.27 ± 0.10	7.13 ± 0.06	7.37 ± 0.05	7.26 ± 0.06	6.32	36	6.61	30	LIM and SH3 domain protein 1	Q14847	16
									6.56	37	PDZ and LIM domain protein 1	O00151	28
109	1.7	2.000E-03	5.97 ± 0.16	6.09 ± 0.15	6.21 ± 0.11	6.21 ± 0.13	6.30	31	6.09	32	Caspase-3	P42574	20
									6.11	34	Syntaxin-11	O75558	12
110	1.7	1.142E-07	8.16 ± 0.07	8.10 ± 0.03	7.92 ± 0.11	7.97 ± 0.07	6.03	24	5.98	23	Heat shock protein beta-1	P04792	24
									6.00	25	Peroxiredoxin-6	P30041	37
111	1.7	4.000E-03	6.48 ± 0.18	6.66 ± 0.11	6.72 ± 0.12	6.72 ± 0.15	5.99	30	6.11	34	Syntaxin-11	O75558	22
112	1.7	7.000E-03	7.00 ± 0.15	7.09 ± 0.08	7.22 ± 0.16	7.11 ± 0.07	5.88	19	5.56	22	Phosphomevalonate kinase	Q15126	36
									5.65; 5.37	21; 21	Ras-related protein Rap-1b; Ras-related protein Rap-1b-like protein	P61224; A6NIZ1	20; 20
113	1.7	1.600E-02	7.86 ± 0.22	7.91 ± 0.11	7.83 ± 0.13	8.06 ± 0.12	5.78	44	5.70	283	Filamin-A	P21333	14
									5.93	89	[FLNA protein]	gi|15779184	45
									5.29	42	Actin, cytoplasmic 1	P60709	23
114	1.7	4.000E-02	7.14 ± 0.11	7.27 ± 0.20	7.16 ± 0.11	7.06 ± 0.13	6.93	15	7.68	18	Peptidyl-prolyl cis-trans isomerase A	P62937	21
115	1.7	2.000E-03	7.32 ± 0.14	7.10 ± 0.19	7.35 ± 0.11	7.31 ± 0.09	6.38	43	6.15	42	Septin-2	Q15019	28
									6.21	43	Septin-5	Q99719	10
116	1.7	1.200E-02	7.06 ± 0.19	7.30 ± 0.14	7.05 ± 0.20	7.23 ± 0.17	4.87	11			unidentified		
117	1.7	2.000E-03	7.03 ± 0.13	6.81 ± 0.12	6.98 ± 0.09	6.86 ± 0.14	5.03	26			unidentified		
118	1.7	7.000E-03	7.15 ± 0.11	6.98 ± 0.16	6.98 ± 0.15	6.93 ± 0.10	4.64	64	4.79	43	UV excision repair protein RAD23 homolog B	P54727	15
119	1.7	3.500E-02	6.58 ± 0.13	6.78 ± 0.20	6.68 ± 0.19	6.78 ± 0.10	6.11	20	5.85	24	Ras-related protein Rab-14	P61106	50
120	1.6	9.054E-06	7.47 ± 0.07	7.66 ± 0.08	7.45 ± 0.07	7.60 ± 0.12	6.23	16	8.22	19	Cofilin-1	P23528	27
									6.30	18	Low molecular weight phosphotyrosine protein phosphatase	P24666	25
									6.20	17	Prefoldin subunit 2	Q9UHV9	38
121	1.6	3.000E-03	7.15 ± 0.08	7.30 ± 0.13	7.19 ± 0.13	7.35 ± 0.13	5.92	34	6.52	76	Fermitin family homolog 3	Q86UX7	9
122	1.6	4.000E-03	7.30 ± 0.09	7.11 ± 0.09	7.30 ± 0.18	7.32 ± 0.13	5.59	26	5.86	31	Peroxiredoxin-4	Q13162	37
									8.26	30	Putative ATP-dependent Clp protease proteolytic subunit, mitochondrial	Q16740	23

123	1.6	3.600E-05	6.85 ± 0.05	6.94 ± 0.07	7.04 ± 0.11	7.05 ± 0.10	6.12	62	7.65	68	Tyrosine-protein phosphatase non-receptor type 6	P29350	6
									7.10	60	Proto-oncogene tyrosine-protein kinase Src	P12931	13
124	1.6	2.000E-03	6.46 ± 0.12	6.27 ± 0.07	6.45 ± 0.13	6.32 ± 0.13	4.68	21			unidentified		
125	1.6	6.126E-04	6.70 ± 0.08	6.90 ± 0.10	6.87 ± 0.12	6.78 ± 0.09	6.36	58	8.54	57	Fibrinogen beta chain	P02675	23
									6.01	58	T-complex protein 1 subunit beta	P78371	12
									7.14	57	Succinyl-CoA:3-ketoacid-coenzyme A transferase 1, mitochondrial	P55809	10
									8.03	57	Cytosol aminopeptidase	P28838	11
126	1.6	3.200E-02	6.35 ± 0.16	6.57 ± 0.13	6.44 ± 0.18	6.42 ± 0.13	5.40	20	5.64; 6.12	25; 24	Ras-related protein Rab-11B; Ras-related protein Rab-11A	Q15907; P62491	19; 19
									6.43	24	Ras-related protein R-Ras	P10301	22
127	1.6	1.000E-03	7.25 ± 0.08	7.19 ± 0.13	7.40 ± 0.11	7.31 ± 0.08	6.55	35	6.56	37	PDZ and LIM domain protein 1	O00151	11
128	1.6	2.000E-02	7.46 ± 0.11	7.33 ± 0.20	7.39 ± 0.08	7.27 ± 0.07	6.68	30	6.11	34	Syntaxin-11	O75558	28
129	1.6	3.700E-02	6.64 ± 0.16	6.59 ± 0.10	6.56 ± 0.16	6.45 ± 0.10	4.72	15	6.11	34	Syntaxin-11	O75558	14
130	1.6	2.800E-02	6.31 ± 0.09	6.28 ± 0.17	6.42 ± 0.12	6.22 ± 0.13	6.12	22	5.64; 6.12	25; 24	Ras-related protein Rab-11B; Ras-related protein Rab-11A	Q15907; P62491	13; 13
131	1.6	3.925E-04	7.89 ± 0.11	8.09 ± 0.09	7.91 ± 0.17	8.09 ± 0.09	5.54	11	5.85	12	Protein S100-A4	P26447	36
132	1.6	1.400E-02	7.06 ± 0.18	7.10 ± 0.10	6.91 ± 0.08	6.98 ± 0.10	4.99	32	5.23	34	Alpha-soluble NSF attachment protein	P54920	22
133	1.6	2.000E-03	6.47 ± 0.14	6.52 ± 0.12	6.53 ± 0.10	6.68 ± 0.08	5.00	15			unidentified		
134	1.6	2.400E-02	6.90 ± 0.14	6.93 ± 0.12	6.75 ± 0.12	6.83 ± 0.13	6.08	25	8.34	32	Enoyl-CoA hydratase, mitochondrial	P30084	28
									6.00	25	Peroxiredoxin-6	P30041	25
135	1.6	1.300E-02	7.67 ± 0.11	7.78 ± 0.15	7.61 ± 0.07	7.60 ± 0.13	5.62	72	5.92	71	Serum albumin	P02768	17
									5.48	70	Heat shock 70 kDa protein 1	P08107	12
									5.37	71	Heat shock cognate 71 kDa protein	P11142	10
136	1.5	7.472E-06	7.41 ± 0.03	7.41 ± 0.09	7.43 ± 0.05	7.24 ± 0.10	5.77	53	5.37	52	Fibrinogen gamma chain	P02679	9
									5.66	53	Cytosolic non-specific dipeptidase	Q96KP4	14
137	1.5	1.000E-03	7.53 ± 0.11	7.66 ± 0.08	7.46 ± 0.09	7.53 ± 0.08	5.64	72	5.92	71	Serum albumin	P02768	14
138	1.5	4.200E-02	6.82 ± 0.18	6.73 ± 0.22	6.72 ± 0.14	6.93 ± 0.07	6.35	73	6.10	61	T-complex protein 1 subunit gamma	P49368	6
									6.11	67	Syntaxin-binding protein 2	Q15833	12
									7.06	74	Succinate dehydrogenase [ubiquinone] flavoprotein subunit, mitochondrial	P31040	10
139	1.5	5.259E-05	7.01 ± 0.07	7.20 ± 0.06	7.06 ± 0.10	7.04 ± 0.06	6.18	25	5.36	32	F-actin-capping protein subunit beta	P47756	22
									6.35	28	Protein ETHE1, mitochondrial	O95571	20
140	1.5	1.600E-02	7.04 ± 0.11	6.93 ± 0.17	7.13 ± 0.10	7.04 ± 0.08	4.97	26	5.26	22	Tumor protein D54	O43399	23
141	1.5	3.429E-04	7.88 ± 0.06	8.02 ± 0.07	7.90 ± 0.09	8.05 ± 0.11	5.56	16	8.41	23	Transgelin-2	P37802	24
									5.47	16	Actin-related protein 2/3 complex subunit 5	O15511	54
142	1.5	5.741E-04	7.53 ± 0.06	7.33 ± 0.13	7.49 ± 0.07	7.40 ± 0.10	4.86	30	5.02	30	Microtubule-associated protein RP/EB family member 1	Q15691	40
									5.09	27	Chloride intracellular channel protein 1	O00299	26

143	1.5	1.100E-02	7.15 ± 0.11	7.20 ± 0.06	7.30 ± 0.12	7.12 ± 0.13	6.27	61	7.10	60	Proto-oncogene tyrosine-protein kinase Src	P12931	16
									8.54	57	Fibrinogen beta chain	P02675	9
144	1.5	1.672E-06	8.35 ± 0.05	8.52 ± 0.05	8.36 ± 0.05	8.41 ± 0.08	5.67	88	5.70	283	Filamin-A	P21333	13
									5.93	89	[FLNA protein]	gi|15779184	41
									5.90	86	Gelsolin	P06396	22
									5.75	84	Coagulation factor XIII A chain	P00488	6
145	1.5	4.300E-02	7.86 ± 0.11	7.68 ± 0.14	7.83 ± 0.15	7.76 ± 0.14	4.50	42	4.73	35	SPARC	P09486	33
									5.29; 5.31	42; 42	Actin, cytoplasmic 1; Actin, cytoplasmic 2	P60709; P63261	14; 14
146	1.5	2.000E-03	8.38 ± 0.07	8.48 ± 0.10	8.48 ± 0.06	8.55 ± 0.10	4.53	16	4.71; 4.67	20; 20	Myosin regulatory light chain MRLC2; Myosin regulatory light chain MRLC3	O14950; P19105	24; 24
									4.80	20	Myosin regulatory light polypeptide 9	P24844	18
147	1.5	7.000E-03	7.99 ± 0.10	7.81 ± 0.12	7.94 ± 0.09	7.93 ± 0.10	6.38	44	6.15	42	Septin-2	Q15019	17
									6.21	43	Septin-5	Q99719	9
148	1.5	8.000E-03	6.60 ± 0.11	6.62 ± 0.07	6.73 ± 0.08	6.53 ± 0.15	4.75	36	4.84	33	Thioredoxin-like protein 1	O43396	27
									5.89	53	Clusterin	P10909	11
149	1.5	1.000E-02	7.37 ± 0.09	7.27 ± 0.12	7.29 ± 0.09	7.19 ± 0.10	5.69	53	5.71	37	L-lactate dehydrogenase B chain	P07195	20
									5.72	58	Histidyl-tRNA synthetase, cytoplasmic	P12081	18
									5.85	50	Rho GTPase-activating protein 1	Q07960	11
150	1.5	4.509E-04	7.30 ± 0.07	7.12 ± 0.13	7.31 ± 0.07	7.17 ± 0.10	6.22	37	6.26	34	Crk-like protein	P46109	29
									6.56	37	PDZ and LIM domain protein 1	O00151	19
151	1.5	4.000E-03	7.29 ± 0.08	7.12 ± 0.11	7.23 ± 0.10	7.18 ± 0.09	4.71	18	4.88	22	Protein LZIC	Q8WZA0	30
152	1.5	2.900E-02	7.13 ± 0.10	6.99 ± 0.08	7.02 ± 0.16	7.14 ± 0.14	6.08	79	6.08	68	Moesin	P26038	15
									5.92	71	Serum albumin	P02768	10
									5.63	93	Caldesmon	Q05682	11
153	1.5	5.000E-03	7.81 ± 0.08	7.65 ± 0.14	7.83 ± 0.07	7.76 ± 0.10	5.59	28	5.36	32	F-actin capping protein subunit beta	P47756	24
									5.66	30	N(G).N(G)-dimethylarginine dimethylaminohydrolase 2	O95865	22
154	1.5	7.532E-04	7.06 ± 0.07	6.93 ± 0.11	7.03 ± 0.08	6.89 ± 0.08	5.25	24	5.36	32	F-actin capping protein subunit beta	P47756	16
									5.69	30	FGFR1 oncogene partner 2	Q9NVK5	21
155	1.5	2.000E-03	7.21 ± 0.11	7.32 ± 0.07	7.15 ± 0.11	7.30 ± 0.09	6.07	12	6.07	10	BolA-like protein 2	Q9H3K6	18
156	1.4	3.000E-03	7.03 ± 0.05	6.95 ± 0.09	7.11 ± 0.09	7.03 ± 0.09	6.32	37	6.61	30	LIM and SH3 domain protein 1	Q14847	18
									6.06	34	Biliverdin reductase A	P53004	15
									6.44	38	GRB2-related adapter protein 2	O75791	17
157	1.4	2.000E-03	8.27 ± 0.08	8.19 ± 0.09	8.11 ± 0.08	8.12 ± 0.11	4.63	16	4.80	20	Myosin regulatory light polypeptide 9	P24844	45

158	1.4	1.700E-02	6.83 ± 0.09	6.70 ± 0.10	6.85 ± 0.13	6.77 ± 0.07	5.41	23	5.56	31	Apolipoprotein A-I	P02647	16
									5.79	25	Eukaryotic translation initiation factor 4E	P06730	26
159	1.4	2.800E-02	6.78 ± 0.16	6.92 ± 0.06	6.75 ± 0.18	6.75 ± 0.10	4.75	20	5.49	82	Calpain-1 catalytic subunit	P07384	5
160	1.4	4.000E-03	7.66 ± 0.10	7.67 ± 0.07	7.81 ± 0.12	7.73 ± 0.07	5.14	19	5.65	21	Ras-related protein Rap-1b	P61224	19
161	1.4	2.400E-02	6.65 ± 0.08	6.48 ± 0.12	6.55 ± 0.10	6.56 ± 0.12	4.88	27	5.16	31	Inositol monophosphatase	P29218	14
162	1.4	4.100E-02	6.74 ± 0.20	6.92 ± 0.11	6.87 ± 0.11	6.89 ± 0.10	6.14	49	6.11	51	Rab GDP dissociation inhibitor beta	P50395	21
									5.87	53	Bleomycin hydrolase	Q13867	11
163	1.4	2.000E-03	7.15 ± 0.08	7.31 ± 0.07	7.16 ± 0.11	7.23 ± 0.08	5.84	25	6.77	29	Endoplasmic reticulum protein ERp29	P30040	36
									4.94	105	AP-1 complex subunit beta-1	Q10567	8
									4.52	54	[unnamed protein product]	gi|194377420	16
164	1.4	8.663E-04	7.28 ± 0.04	7.33 ± 0.08	7.18 ± 0.09	7.25 ± 0.07	5.90	16	8.22	19	Cofilin-1	P23528	47
									8.41	23	Transgelin-2	P37802	20
165	1.4	1.900E-02	6.45 ± 0.07	6.45 ± 0.11	6.44 ± 0.14	6.59 ± 0.11	5.99	22	6.12	24	Ras-related protein Rab-11A	P62491	43
166	1.4	4.300E-02	7.49 ± 0.08	7.35 ± 0.09	7.42 ± 0.12	7.47 ± 0.14	5.39	19	5.65	21	Ras-related protein Rap-1b	P61224	26
167	1.4	1.200E-02	7.05 ± 0.09	7.10 ± 0.11	6.96 ± 0.10	6.98 ± 0.05	5.53	62	5.95	63	Dihydropyrimidinase-related protein 2	Q16555	37
									5.98	57	Protein disulfide-isomerase A3	P30101	15
168	1.4	7.000E-03	7.57 ± 0.05	7.57 ± 0.09	7.70 ± 0.09	7.61 ± 0.09	4.65	18	5.12	19	Galectin-related protein	Q3ZCW2	30
169	1.4	7.000E-03	6.35 ± 0.08	6.24 ± 0.13	6.35 ± 0.05	6.21 ± 0.12	5.54	38	6.26	42	Beta-parvin	Q9HBI1	10
170	1.4	7.000E-03	8.20 ± 0.09	8.31 ± 0.06	8.17 ± 0.09	8.19 ± 0.10	5.71	72	5.92	71	Serum albumin	P02768	29
									5.37	71	Heat shock cognate 71 kDa protein	P11142	16
171	1.3	8.555E-04	7.35 ± 0.09	7.25 ± 0.05	7.38 ± 0.05	7.26 ± 0.08	5.34	30	5.44	28	Proteasome activator complex subunit 2	Q9UL46	19
172	1.3	4.800E-02	6.70 ± 0.11	6.65 ± 0.09	6.77 ± 0.09	6.75 ± 0.09	5.45	24	8.15	139	Multimerin-1	Q13201	3
173	1.3	3.800E-02	7.16 ± 0.10	7.07 ± 0.09	7.04 ± 0.09	7.03 ± 0.09	4.87	29	5.26	22	Tumor protein D54	O43399	53
									5.16	31	Inositol monophosphatase	P29218	18
174	1.3	3.900E-02	7.41 ± 0.09	7.48 ± 0.07	7.40 ± 0.12	7.52 ± 0.11	5.81	12	5.26	22	Tumor protein D54	O43399	23
175	1.3	2.500E-02	6.65 ± 0.09	6.75 ± 0.11	6.63 ± 0.09	6.73 ± 0.08	6.04	60	5.98	57	Protein disulfide-isomerase A3	P30101	12
									5.87	56	Tripartite motif-containing protein 58	Q8NG06	9
									8.54	57	Fibrinogen beta chain	P02675	14
									5.58	59	Serine/threonine-protein phosphatase 2B catalytic subunit alpha isoform	Q08209	8
176	1.3	3.000E-03	7.53 ± 0.03	7.40 ± 0.06	7.45 ± 0.08	7.48 ± 0.08	4.65	21	4.84	20	Translationally-controlled tumor protein	P13693	34
177	1.3	2.000E-02	7.44 ± 0.08	7.42 ± 0.09	7.38 ± 0.08	7.32 ± 0.09	5.31	62	5.42	60	T-complex protein 1 subunit theta	P50990	33
									5.29; 5.31	42; 42	Actin, cytoplasmic 1; Actin, cytoplasmic 2	P60709; P63261	28; 28
									5.45	60	T-complex protein 1 subunit epsilon	P48643	13

178	1.3	4.000E-03	7.59 ± 0.09	7.59 ± 0.07	7.52 ± 0.08	7.48 ± 0.05	5.25	35	5.30; 5.21	36; 36	Serine/threonine-protein phosphatase 2A catalytic subunit alpha isoform; Serine/threonine-protein phosphatase 2A catalytic subunit beta isoform	P67775; P62714	17; 17
									5.45	33	F-actin capping protein subunit alpha-1	P52907	20
179	1.3	3.300E-02	7.10 ± 0.07	7.17 ± 0.08	7.20 ± 0.11	7.09 ± 0.10	5.24	63	5.20	56	Ubiquitin carboxyl-terminal hydrolase 14	P54578	15
180	1.3	4.200E-02	7.46 ± 0.09	7.57 ± 0.09	7.46 ± 0.11	7.47 ± 0.09	5.81	105	5.77	272	Talin-1	Q9Y490	15
									5.74	107	Neutral alpha-glucosidase AB	Q14697	17
									5.70	283	Filamin-A	P21333	6
									5.93	89	[FLNA protein]	gi|15779184	21
181	1.3	3.100E-02	7.27 ± 0.09	7.34 ± 0.08	7.24 ± 0.12	7.35 ± 0.07	5.97	12			unidentified		
182	1.3	3.500E-02	7.41 ± 0.08	7.35 ± 0.07	7.36 ± 0.07	7.45 ± 0.09	5.38	26	5.45	29	Chloride intracellular channel protein 4	Q9Y696	20
183	1.3	1.400E-02	7.36 ± 0.08	7.29 ± 0.11	7.39 ± 0.05	7.40 ± 0.03	5.59	27	5.36	32	F-actin capping protein subunit beta	P47756	16
									5.66	30	N(G).N(G)-dimethylarginine dimethylaminohydrolase 2	O95865	30
184	1.3	1.700E-02	7.17 ± 0.07	7.07 ± 0.07	7.16 ± 0.07	7.09 ± 0.09	5.65	41	5.71	37	L-lactate dehydrogenase B chain	P07195	18
									6.02	45	Guanine nucleotide-binding protein subunit beta-5	O14775	12
185	1.3	1.000E-03	8.19 ± 0.05	8.12 ± 0.06	8.20 ± 0.06	8.22 ± 0.03	5.75	56	5.98	57	Protein disulfide-isomerase A3	P30101	34
186	1.3	3.000E-03	7.84 ± 0.05	7.77 ± 0.04	7.87 ± 0.06	7.79 ± 0.07	5.63	50	5.61	48	Actin-related protein 3	P61158	34
187	1.2	3.000E-02	7.15 ± 0.06	7.06 ± 0.06	7.13 ± 0.09	7.13 ± 0.05	5.95	15	6.13	17	Ubiquitin-conjugating enzyme E2 N	P61088	42
188	1.2	1.000E-02	8.57 ± 0.05	8.66 ± 0.05	8.60 ± 0.05	8.61 ± 0.06	5.78	88	5.90	86	Gelsolin	P06396	21
									5.70	283	Filamin-A	P21333	8
									5.93	89	[FLNA protein]	gi|15779184	28
									5.75	84	Coagulation factor XIII A chain	P00488	14
189	1.2	2.700E-02	8.21 ± 0.05	8.13 ± 0.05	8.21 ± 0.05	8.18 ± 0.09	5.38	35	5.45	33	F-actin capping protein subunit alpha-1	P52907	26
									5.60	38	Guanine nucleotide-binding protein G(I)/G(S)/G(T) subunit beta-1	P62873	19
									5.60	38	Guanine nucleotide-binding protein G(I)/G(S)/G(T) subunit beta-2	P62879	19
									5.60	38	Guanine nucleotide-binding protein subunit beta-4	Q9HAV0	14
190	1.2	2.600E-02	7.49 ± 0.07	7.41 ± 0.04	7.48 ± 0.08	7.50 ± 0.05	5.92	23	5.89	25	Growth factor receptor-bound protein 2	P62993	23

Log(V_N) – logarithm of spot normalized volume; N – resting platelets; A – arachidonic acid activation; C – collagen activation; T – thrombin activation; AN – accession number (SWISS-PROT and NCBInr); SC – sequence coverage of protein

Table II. Top ten spot expression profile groups.

Group	Spot	Experimental pI	Experimental Mw (kDa)	Calculated pI	Calculated Mw (kDa)	Protein identification	AN
Group 1 A++T+	4	5.52	94	5.70	283	Filamin-A	P21333
				5.93	89	[FLNA protein]	gi|15779184
				5.90	86	Gelsolin	P06396
	21	5.14	16	8.41	23	Transgelin-2	P37802
	27	5.58	90	5.70	283	Filamin-A	P21333
				5.93	89	[FLNA protein]	gi|15779184
				5.90	86	Gelsolin	P06396
	28	5.54	60	5.95	63	Dihydropyrimidinase-related protein 2	Q16555
	65	5.62	17	8.41	23	Transgelin-2	P37802
Group 2 A++	10	5.55	107	5.77	272	Talin-1	Q9Y490
	13	5.52	107	5.77	272	Talin-1	Q9Y490
				8.54	57	Fibrinogen beta chain	P02675
	17	5.10	124	5.50	228	Myosin-9	P35579
				5.29	42	Actin, cytoplasmic 1	P60709
				6.39	128	Integrin alpha-6	P23229
	24	5.65	111	5.77	272	Talin-1	Q9Y490
	44	5.77	100	5.77	272	Talin-1	Q9Y490
				5.70	283	Filamin-A	P21333
				5.93	89	[FLNA protein]	gi|15779184
	58	5.68	108	5.77	272	Talin-1	Q9Y490
	66	5.76	114	5.77	272	Talin-1	Q9Y490
				5.70	283	Filamin-A	P21333
				5.58	113	Ubiquitin-like modifier-activating enzyme 7	P41226
Group 3 T--	11	6.76	53	8.54	57	Fibrinogen beta chain	P02675
	30	5.35	52	5.37	52	Fibrinogen gamma chain	P02679
	38	5.49	52	5.37	52	Fibrinogen gamma chain	P02679
	61	6.50	58	8.54	57	Fibrinogen beta chain	P02675
	94	5.63	52	5.61	48	Actin-related protein 3	P61158
				5.37	52	Fibrinogen gamma chain	P02679
Group 4 T-	5	6.90	72	6.17	67	WD repeat-containing protein 1	O75083
	6	6.99	71	6.17	67	WD repeat-containing protein 1	O75083
				6.40	63	Stress-induced-phosphoprotein 1	P31948
	26	6.84	73	7.23	81	Glycerol-3-phosphate dehydrogenase, mitochondrial	P43304
	40	6.85	57	8.54	57	Fibrinogen beta chain	P02675
	71	6.78	74	7.23	81	Glycerol-3-phosphate dehydrogenase, mitochondrial	P43304

Group 5 A--T-	47	6.61	35	6.56	37	PDZ and LIM domain protein 1	O00151
	49	6.35	86	6.22	62	Zyxin	Q15942
				6.81	79	Serotransferrin	P02787
	59	6.85	34	6.56	37	PDZ and LIM domain protein 1	O00151
	67	6.59	40	6.37	40	Twinfilin-2	Q6IBS0
				6.56	37	PDZ and LIM domain protein 1	O00151
	73	6.66	34	6.70	40	Aflatoxin B1 aldehyde reductase member 2	O43488
				6.56	37	PDZ and LIM domain protein 1	O00151
	74	6.56	86	6.81	79	Serotransferrin	P02787
				6.22	62	Zyxin	Q15942
	84	6.40	36	6.56	37	PDZ and LIM domain protein 1	O00151
	89	6.42	37	6.26	34	Crk-like protein	P46109
	97	6.79	39	6.37	40	Twinfilin-2	Q6IBS0
				6.16	40	Mannose-1-phosphate guanyltransferase beta	Q9Y5P6
	108	6.32	36	6.61	30	LIM and SH3 domain protein 1	Q14847
				6.56	37	PDZ and LIM domain protein 1	O00151

Group 6 A-	33	4.78	75	5.14	47	Serum deprivation-response protein	O95810
	37	4.81	72	5.14	47	Serum deprivation-response protein	O95810
				5.29; 5.31	42; 42	Actin, cytoplasmic 1; Actin, cytoplasmic 2	P60709; P63261
	41	6.34	86	6.22	62	Zyxin	Q15942
				8.54	57	Fibrinogen beta chain	P02675
	50	6.18	88	5.75	84	Coagulation factor XIII A chain	P00488
	54	6.23	87	5.75	84	Coagulation factor XIII A chain	P00488
	103	6.31	43	6.15	42	Septin-2	Q15019
				5.31	42	Actin. cytoplasmic 2	P63261
				8.50	40	Pleckstrin	P08567
	147	6.38	44	6.15	42	Septin-2	Q15019
				6.21	43	Septin-5	Q99719

Group 7 N-	60	5.75	23	7.67	28	Thioredoxin-dependent peroxide reductase, mitochondrial	P30048
	109	6.30	31	6.09	32	Caspase-3	P42574
				6.11	34	Syntaxin-11	O75558
	111	5.99	30	6.11	34	Syntaxin-11	O75558
	123	6.12	62	7.65	68	Tyrosine-protein phosphatase non-receptor type 6	P29350
				7.10	60	Proto-oncogene tyrosine-protein kinase Src	P12931

Group 8 A+T+	15	6.00	16	8.67	25	Vesicle-trafficking protein SEC22b	O75396
				8.41	23	Transgelin-2	P37802
	18	5.93	14	6.56	37	PDZ and LIM domain protein 1	O00151
	19	5.53	14			unidentified	
	32	6.06	16	6.32; 6.84	21; 21	ADP-ribosylation factor 1; ADP-ribosylation factor 3	P84077; P61204
	35	6.92	30	8.27	52	Adenylyl cyclase-associated protein 1	Q01518
				4.71	133	Thrombospondin-1	P07996
				6.95	24	[Thrombospondin-1 N-Terminal Domain]	gi|88191913
				6.54	32	S-formylglutathione hydrolase	P10768
	45	5.13	13			unidentified	
	46	5.61	14			unidentified	
	48	5.81	19	5.64; 6.12	25; 24	Ras-related protein Rab-11B; Ras-related protein Rab-11A	Q15907; P62491
	78	5.30	27	5.36	32	F-actin capping protein subunit beta	P47756

Group 9 A+T+	29	5.80	36	6.52	76	Fermitin family homolog 3	Q86UX7
	53	5.44	93	5.50	124	Vinculin	P18206
				5.90	86	Gelsolin	P06396
	62	4.62	12	4.67	29	Tropomyosin alpha-4 chain	P67936
	121	5.92	34	6.52	76	Fermitin family homolog 3	Q86UX7
	141	5.56	16	8.41	23	Transgelin-2	P37802
				5.47	16	Actin-related protein 2/3 complex subunit 5	O15511

Group 10 T++	1	6.36	29	4.71	133	Thrombospondin-1	P07996
				6.95	24	[Thrombospondin-1 N-Terminal Domain]	gi|88191913
	9	5.88	30	4.71	133	Thrombospondin-1	P07996
				8.00	28	[Thrombospondin-1 N-Terminal Domain]	gi|88191917
				5.37	52	Fibrinogen gamma chain	P02679
	79	5.99	29	4.71	133	Thrombospondin-1	P07996
				6.95	24	[Thrombospondin-1 N-Terminal Domain]	gi|88191913
	86	5.52	32	4.71	133	Thrombospondin-1	P07996
				6.95	24	[Thrombospondin-1 N-Terminal Domain]	gi|88191913
				6.11	34	Syntaxin-11	O75558

Ten spot expression profile groups were chosen as a result of the correlation analysis (the dendrogram tree). All groups were limited to have Distance value less than 0.5. Each expression group contains spots with similar expression profiles (highly correlated spots); Platelet activation group A – AA, C – collagen, T – thrombin, N - no activation; Minus or plus sign putative spot under- or over-expression (e.a. A++ shows a strong overexpression of all spots in the entire expression profile group in platelets activated by AA, while no over- or under-expression occurs in other activation groups); AN – accession number (SWISS-PROT and NCBInr)
