# Supplementary figures and images for: Proteome changes in platelets activated by arachidonic acid, collagen, and thrombin
Source: Proteome Sci. 2010 Nov 12;8:56. doi: 10.1186/1477-5956-8-56 (PMC2996359; doi:10.1186/1477-5956-8-56)

Dendrogram

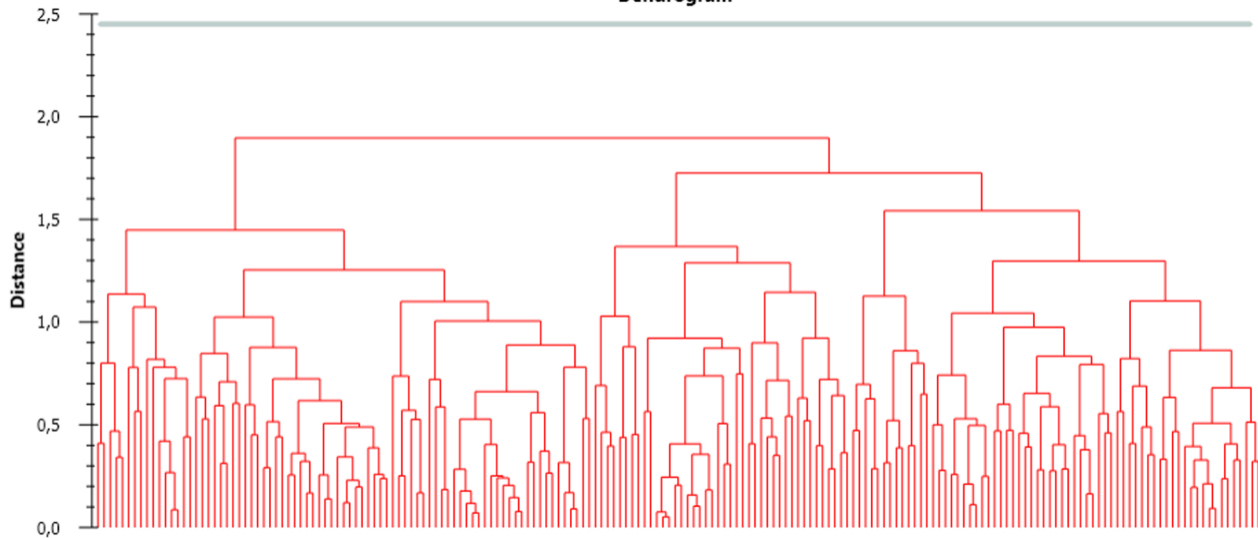

Supplement: Additional file 2 — Figure 6 - The dendrogram tree. The dendrogram tree - a visual representation of the spot correlation data of all spots that were found to be significantly different among all four platelet groups (resting and activated platelets) when mutually compared. [file 1477-5956-8-56-S2.PDF]

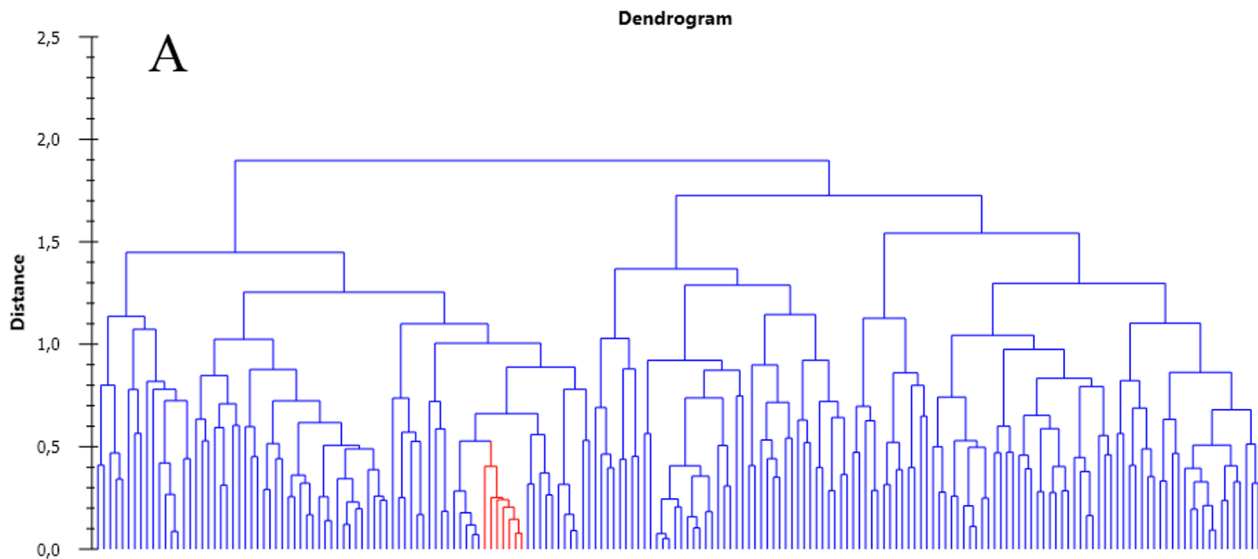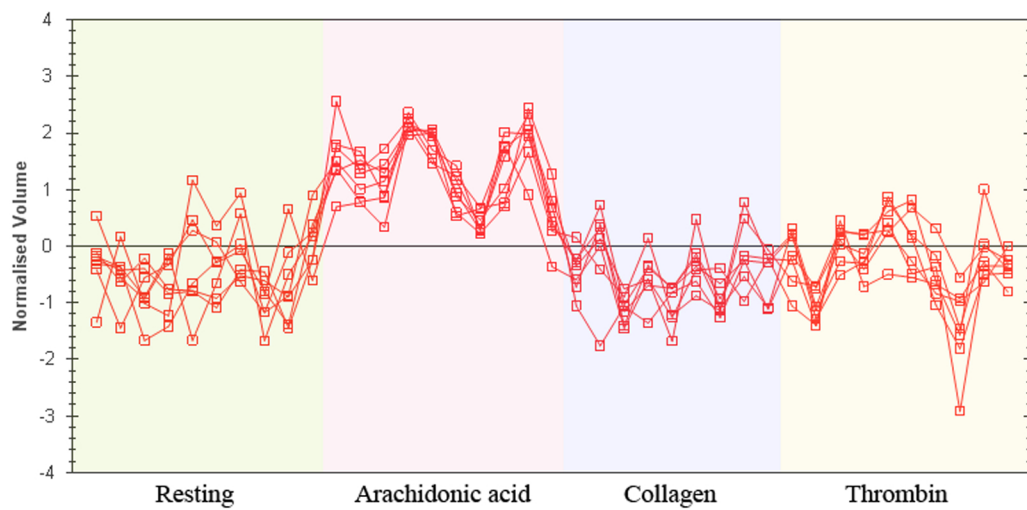

Supplement: Additional file 3 — Figure 7 - An example of the expression profile group (group 2). A - The dendrogram tree with highlighted (in red colour) spots of spot expression profile group 2. The x-axis is composed of all 190 spots that significantly differed when resting and by different agonist activated platelet proteomes were mutually compared. Spots are grouped by their expression patterns using correlation analysis and hierarchical clustering.; B - The expression profile of spot expression profile group 2 spots. All spots for each activation group (resting platelets and by arachidonic acid, collagen, and thrombin activated platelets as indicated below the x-axis) are displayed. [file 1477-5956-8-56-S3.PDF]
